# Moving into Protected Areas? Setting Conservation Priorities for Romanian Reptiles and Amphibians at Risk from Climate Change

**DOI:** 10.1371/journal.pone.0079330

**Published:** 2013-11-04

**Authors:** Viorel D. Popescu, Laurenţiu Rozylowicz, Dan Cogălniceanu, Iulian Mihăiţă Niculae, Adina Livia Cucu

**Affiliations:** 1 Earth to Ocean Research Group, Department of Biological Sciences, Simon Fraser University, Burnaby, British Columbia, Canada; 2 Centre for Environmental Research (CCMESI), University of Bucharest, Bucharest, Romania; 3 Faculty of Natural and Agricultural Sciences, University Ovidius Constanţa, Constanţa, Romania; Swedish University of Agricultural Sciences, Sweden

## Abstract

Rapid climate change represents one of the top threats to biodiversity, causing declines and extinctions of many species. Range shifts are a key response, but in many cases are incompatible with the current extent of protected areas. In this study we used ensemble species distribution models to identify range changes for 21 reptile and 16 amphibian species in Romania for the 2020s and 2050s time horizons under three emission scenarios (A1B = integrated world, rapid economic growth, A2A = divided world, rapid economic growth [realistic scenario], B2A = regional development, environmentally-friendly scenario) and no- and limited-dispersal assumptions. We then used irreplaceability analysis to test the efficacy of the Natura 2000 network to meet conservation targets. Under all scenarios and time horizons, 90% of the species suffered range contractions (greatest loses under scenarios B2A for 2020s, and A1B for 2050s), and four reptile species expanded their ranges. Two reptile and two amphibian species are predicted to completely lose climate space by 2050s. Currently, 35 species do not meet conservation targets (>40% representation in protected areas), but the target is predicted to be met for 4 - 14 species under future climate conditions, with higher representation under the limited-dispersal scenario. The Alpine and Steppic-Black Sea biogeographic regions have the highest irreplaceability value, and act as climate refugia for many reptiles and amphibians. The Natura 2000 network performs better for achieving herpetofauna conservation goals in the future, owing to the interaction between drastic range contractions, and range shifts towards existing protected areas. Thus, conservation actions for herpetofauna in Romania need to focus on: (1) building institutional capacity of protected areas in the Alpine and Steppic-Black Sea biogeographic regions, and (2) facilitating natural range shifts by improving the conservation status of herpetofauna outside protected areas, specifically in traditionally-managed landscapes and abandoned cropland.

## Introduction

Protected areas continue to be one of the main instruments in the biodiversity conservation toolbox, and the total area benefiting of legal protection has been increasing worldwide [1]. Between 10.1% and 15.5% of land area is under some form of protection worldwide [2], and at a global level protected areas have been established at a fast pace, overcoming our capacity to manage them [1]. Despite concerted efforts towards designing resilient protected areas, and implementing ecologically-relevant conservation strategies, reserve networks still fail to conserve important biodiversity elements (species- to biome-level misrepresentation) [3]. Adding to these issues, the world’s current protected areas network faces additional challenges from climate change [4]. Particularly, identifying of priority areas within and outside the current protected network that are resilient to climate change is challenged by uncertainties surrounding future climate projections and species responses to climate change [5-7]. 

Climate change has already caused declines or extinctions of many amphibian and reptile species through direct effects, such as altered thermal niches, or through synergies with other threats, such as pathogens and land use changes [8,9]. A recent evaluation of the status of European herpetofauna has shown that 19% of reptiles and 23% of amphibians are threatened [10,11]. In the context of future climate change, range shifts are a key response, and can affect species representation in protected areas. Species vary in their dispersal potential, and limited dispersal may hinder the ability of species to track potentially suitable climate space [12]. Ectotherms are more likely to track their climate space compared to endotherms [13], and major shifts in herpetofaunal assemblages caused by climate change are predicted worldwide [14]. Thus, from a practical conservation perspective, it is critical to evaluate potential spatial mismatches between predicted range shifts of threatened reptiles and amphibians and the extent of protected areas, and evaluate emerging conservation challenges (e.g., loser species) and opportunities (e.g., winner species; sensu [15]).

The pan-European Natura 2000 protected areas network is projected as a safety net for conserving priority species and habitats. Despite existing design flaws that limit the connectedness and functional connectivity across national borders [16], the Natura 2000 network is the outcome of two of the strongest legal continental-level conservation instruments: the Habitats and Birds Directives. In 2012, the Natura 2000 network covered 17.9% of terrestrial Europe, and it is projected to reach 20% by 2020. The designation of Natura 2000 sites is not always based on quantifiable conservation targets or comprehensive spatial planning (e.g., [17]). As such, many sites are designated simply to achieve area targets set by European Union for country-level protection (i.e., 20% of any EU country protected in Natura 2000 network) [18]. In the context of the enlarged European Union, little attention has been paid to comprehensive conservation planning in Eastern European countries [19]. While European-level studies addressing the issue of protected areas resilience in the face of climate change exist (e.g., [15,20]), the coarse resolution of such analyses may not be useful for national-level planning. 

The aim of our study is to identify conservation priorities for Romania’s amphibians and reptiles in the face of climate change, using a spatial conservation planning approach. We make use of a comprehensive species occurrence dataset, which includes 19 amphibian and 23 reptile species found in Romania; of these, 10 amphibian (52.6%) and 13 reptiles species (56.6%) are at the limit of their geographical ranges, which increases the uncertainties for their effective conservation in the light of predicted climate-induced range shifts [21]. Using ensemble forecasting species distribution models, we evaluated how climate change and dispersal ability shape future amphibians and reptiles distributions in Romania. We then assessed species representation in the Natura 2000 network under current, as well as future climate conditions. We tested the efficacy of Natura 2000 sites for achieving European conservation targets, and identified priority areas (irreplaceability hotspots) within European biogeographic regions. We then evaluated the strength of spatial association between the existing Natura 2000 network and predicted priority areas for conservation (under current and future climate scenarios) to identify potential gaps, as well as opportunities for conservation within the current protected areas network. 

## Materials and Methods

### Species data

For this analysis we used occurrence only data for 21 species of reptiles and 16 amphibians from the first nation-wide database of species occurrences of any taxa in Romania [22,23]. This database reports the location of actual occurrences, and we aggregated the data to presence/absence within 2548 10 × 10 km cells (planning units = PUs, hereafter) based on a Universal Transverse Mercator (UTM) grid. The 10 × 10 km grid represents a standardized way of reporting Natura 2000 effectiveness [24]. According to the European Red List of reptiles [10] and amphibians [11], two amphibian (*Pelobates syriacus* and *Triturus dobrogicus*) and three reptile species (*Elaphe quatuorlineata, Lacerta praticola*, and *Testudo hermanni*) are ranked as Near Threatened, and four reptile species are ranked as Vulnerable (*Emys orbicularis, Eremias arguta, Testudo graeca*, and *Vipera ursinii*). We did not model the distributions of *Rana esculenta* and *Rana ridibunda*, which together represent a species complex difficult to distinguish in the field [25], *Rana dalmatina* and *Eryx jaculus* (both species having low number of occurrence data in our database) and *Natrix natrix* (the only reptile not protected under the Romanian legislation). For this analysis we used binomial nomenclature from EU Habitats Directive and Romanian legislation. 

### Protected areas data

The spatial data on the location/boundaries of the 374 terrestrial Sites of Community Importance (SCI, designated to protect habitats and species listed in EU Habitats Directive) was provided by the Romanian Ministry of Environment (www.mmediu.ro, accessed 6 October 2012). The area covered by terrestrial SCIs in Romania is 40168 km^2^ (16.84% of the territory). We then selected PUs that overlapped with SCIs (i.e., protected PUs) using an iterative aggregation method [4], which yielded a total area of protected PUs equal to the actual area in SCIs. This procedure yielded 410 PUs (16% of all PUs, similar to the proportion of protected areas in SCIs), with a PU in protected status when >36% of its area was contained in SCIs. At European Union level, the effectiveness of Natura 2000 network is estimated based on European biogeographic regions (BGR). Romanian territory lies into five BGRs: Alpine, Continental, Pannonian, Steppic, and Black Sea [17]. For the purpose of this analysis, the Black Sea BGR was merged with the adjacent Steppic BGR due to its limited terrestrial coverage. 

### Environmental data

We derived a set of climate parameters for current conditions from the WorldClim dataset [26], which contains bioclimatic grids for 1950 - 2000 at a 30 arc-second resolution. Based on the life histories and known physiological requirements of our study species, we selected a set of nine bioclimatic variables for modeling their distribution. For amphibians, we used : (1) annual mean temperature, (2) mean diurnal range, (3) isothermality (mean temperature diurnal range/temperature annual range), (4) annual temperature range (5), mean temperature of the wettest quarter (6), precipitation of the warmest quarter, and (7) precipitation of the coldest quarter. For reptiles, we used a slightly different set of bioclimatic variables that included variables (1) – (4), as well as (8) mean annual temperature of the warmest quarter, and (9) annual precipitation. We used several moisture-related variables for amphibians because of their aquatic-terrestrial life-cycles and susceptibility to desiccation, while for reptiles the variables are mostly related to thermoregulation, with less emphasis on moisture availability. We only used variables with pairwise correlation coefficients <0.7. Current climate data was freely available from the WorldClim database (www.worldclim.org; accessed 10 August 2012).

Future climate projections were derived for two time horizons: 1991 - 2020 (2020’s) and 2021 - 2050 (2050’s) and three IPCC emission scenario families (SRES A1, A2, and B2) from the HadCM3 30-arc-second resolution climate model developed by the Climate Research Unit at the University of East Anglia [27]. Specifically, we chose scenarios A1B, A2A and B2A to capture uncertainties around climate change projections: (1) scenario A1B describes a more integrated world, with rapid economic growth and emphasis placed on all energy sources; (2) scenario A2A describes a more divided world, with regionally-oriented economic growth, increased energy and land-use changes (more realistic scenario); (3) scenario B2A describes regionally-oriented development, but with emphasis on environmental protection, and slower land-use changes (more environmentally-friendly scenario). These scenarios capture a wide range of variability in predicted CO_2_ emissions (between 11 Gt/year (B2A) and 18 Gt/year (A2A) by 2050s (*http://www.ipcc.ch/ipccreports/tar/wg1/029.htm*). Future climate data was freely available from the CGIAR Research Program on Climate Change, Agriculture and Food Security (http://www.ccafs-climate.org; accessed 10 August 2012). 

Species distributions are broadly associated to climatic variables, but land use changes shape the current distribution of species at a local scale. Thus, including land cover variables improves the performance of species distribution models [28]. We used the 2006 CORINE land cover dataset (European Environment Agency, Copenhagen, Denmark) to extract the proportion of developed, forested, agricultural, and herbaceous lands within each 10 × 10 km grid cell.

### Species distribution modeling

We modeled species distributions using seven models in the bioclimatic niche modeling package *BIOMOD* [29] implemented in R 2.15.1 [30]. The models included: (1) generalized additive models (GAM), (2) multivariate adaptive regression splines (MARS), (3) classification tree analysis (CTA), (4) artificial neural networks (ANN), (5) generalized boosted regression trees (GBM), (6) random forests (RF), and (7) flexible discriminant analysis (FDA). For each species, we generated pseudo-absences using a random selection among the grid cells where the species was not reported, while maintaining a 50% prevalence [31].

Models were calibrated for the baseline (1950 - 2000) using an 80% random sample of the occurrence data, and model performance was assessed using the remaining 20%. We evaluated model projections between observed and predicted distributions using area under the curve (AUC) of the receiver operating characteristic (ROC) [32], and the true skill statistic (TSS) [31]. Only species with AUC >0.7 [33] and TSS >0.3 have good prediction accuracy, and we found that no species had to be removed. Further, we used ensemble forecasting [34] to compute consensus projections for each species and scenario separately using the weighted average probability of occurrence per grid cell [35]; weights were based on the TSS obtained on the evaluation data. We then transformed the probability of occurrence for each species to presence-absence data by optimizing a threshold that maximizes the percentage of presence and absence correctly predicted for ROC curves [36]. We assessed the contribution of our dependent variables to predicting species ranges using the relative variable importance across all models [29]. The importance of each variable is calculated as 1 minus the correlation between the original prediction and a prediction made with a permuted variable; low correlations between the two predictions (i.e., high values) are indicative of highly influential variables. 

**Figure 1:**
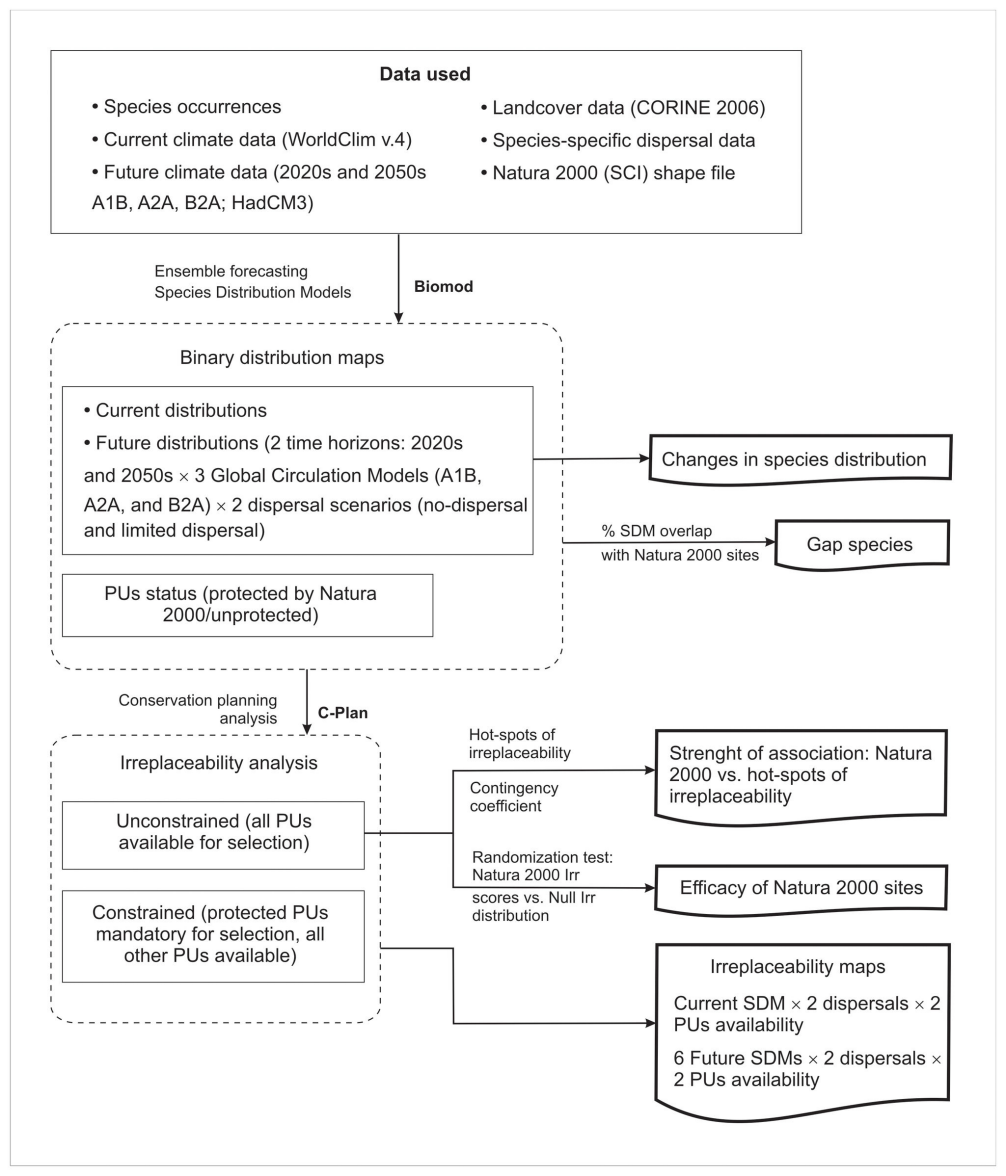
Flowchart of stages of analysis and data inputs.

To account for differences in species dispersal abilities, and to distinguish between areas with future suitable climatic conditions and areas with colonization potential, we incorporated dispersal data available from literature for each species, or for closely related species (i.e., maximum annual dispersal distances; Table S1). The annual dispersal values were then multiplied by 20 and respectively, 50 (i.e., years in the two time horizons) to obtain a maximum dispersal potential. Effective dispersal is influenced by the habitat matrix and potential barriers to movement (e.g., large bodies of water, high traffic highways, agricultural land, urban and rural areas, etc.), and habitat-specific vagility plays a large role in the successful dispersal of both amphibians [37] and reptiles [38]. Because the maximum annual dispersal distances do not account for such impacts, we also considered a more conservative scenario of no dispersal. Thus, for the two scenarios (with and without dispersal) we estimated potential distributional shifts as the difference between the number of grid cells currently occupied and the number predicted to be occupied under climate change (2020s and 2050 time horizons × 3 emission scenarios) (Figure 1; *Changes in species distributions*).

### Conservation value analyses – irreplaceability and gap species

We used irreplaceability analysis [39,40] to evaluate the contribution of current Natura 2000 sites for reaching specific conservation targets defined for amphibians and reptiles, and to identify new priority areas for conservation. We conducted the irreplaceability analysis using C-Plan Systematic Conservation Planning System, version 4.1 [41], and binary (occupied/not occupied) species distribution projections, with and without dispersal under all climate change scenarios and time horizons. Irreplaceability of a PU is defined as its potential contribution to achieving specific conservation targets within a protected areas network [42]. The irreplaceability score reflects the importance of a PU for achieving the nation-wide conservation target [40]. We estimated the irreplaceability score for a given PU*x*, which is calculated by adding the irreplaceability scores for all species predicted to be present within PU*x*. Irreplaceability scores range between interval 0 and 1, where values close to 1 identify planning units that are critical for achieving a given conservation target, and values close to 0 represent PUs easily replaceable sites, not contributing to conservation targets [39]. We ranked the irreplaceability scores into six classes: very low irreplaceability score (≤0.2), low irreplaceability score (0.2 - 0.4), moderate irreplaceability score (0.4 - 0.6), high irreplaceability score (0.6 - 0.8), very high irreplaceability score (0.8 - <1), and completely irreplaceable (score = 1).

Irreplaceability analysis requires defining specific conservation targets for each species. At European level, a species is considered to have excellent conservation status when >60% of its range is included within Natura 2000 sites, and insufficient representation when <20% of the range is protected. We decided to adopt a middle-ground representation threshold, and used a 40% target for each species. For each dispersal scenario (i.e. limited dispersal and no dispersal) and climate change projection we conducted irreplaceability analyses under two PU availability scenarios: (1) unconstrained (SCIs and all other PUs available), and (2) constrained with SCIs mandatory (i.e., irreplaceability score for SCIs = 1, and all other PUs available). The first PU availability scenario was used to simulate a new protected area network, while the second scenario was used for identifying new highly irreplaceable areas to be added to the existing Natura 2000 network (Figure 1; *Irreplaceability maps*). For each dispersal scenario, we ran a total of 14 irreplaceability analyses: two for current distributions (2 PU availability scenarios), and 12 for future distributions ((3 GCM-2020s + 3 GCM-2050s) × 2 PU availability scenarios). 

Finally, we assessed the performance of the current Natura 2000 sites for reaching amphibian and reptile conservation targets by evaluating the percent range overlap with Natura 2000 sites (Figure 1; *Gap species*). Thus, species were classified as: (1) fully covered species (>40% of range is protected) (2), partial gap species (<40% of range is protected), and (3) gap species (no representation in protected areas).

### Natura 2000 effectiveness

For each unconstrained time horizon, we tested the efficacy of SCIs for conserving Romanian amphibian and reptile diversity using a randomization test under the null hypothesis that irreplaceability value of Natura 2000 sites is no different from that of a Null irreplaceability distribution [43] (Figure 1; *Efficacy of Natura 2000 sites*). The Null irreplaceability distributions were generated by using means of irreplaceability scores from 410 grid cells (i.e., equal to the number of protected PUs), drawn randomly from all the PUs pool regardless of their protection status; we repeated the procedure 1000 times for each scenario. 

For the same unconstrained irreplaceability analysis, we performed a hot-spot analysis using the Getis-Ord *Gi* statistic to identify contiguous clusters of cells with irreplaceability values greater than expected [44] (Figure 1; *Strength of association: Natura 2000 vs. hotspots or irreplaceability*). The Getis-Ord *Gi* statistic uses the local matrix of planning units (i.e., adjacent grid cells) to identify aggregations of high and low irreplaceability values by assigning Z-scores to each areal unit (Z-scores >1.96 denote significant hot spots of irreplaceability). We computed Getis-Ord Gi Z-scores in ArcGIS 10 (ESRI, Redlands, CA) using a threshold of 1.5 km, in order to assess each PU in relation to its eight neighboring PUs.

We further evaluated the degree of overlap between protected PUs and irreplaceability hot-spots using the *phi* (*φ*) contingency coefficient. *Phi* takes values between -1 and 1, with values ≤ -0.7 and ≥0.7 indicating a strong association [45]. For each scenario, we built a matrix containing the following combinations: 1) protected PUs and irreplaceability hot-spots; 2) protected PUs and non-hot-spots; 3) unprotected PUs and hot-spots; 4) unprotected PUs and non-hot-spots. The weak correlations can be generated when: (1) the number of irreplaceability hot-spot grid cells is low, and (2) there is complete overlap between between hot-spots and protected PUs. Thus, we consider a significant degree of similarity between hot-spots and Natura 2000 sites, when: (1) φ >0.3 or (2) percent overlap >50% [45].

## Results

### Changes in species distributions

The seven modeling techniques performed well for all species, and median AUC values ranged between 0.885 and 0.952 across models (Table S2). Random Forests provided the best predictive performance, with AUC >0.95 for 12 amphibian and 9 reptile species, followed by Generalized Boosted Regression Trees and Generalized Additive Models, while Classification Tree Analysis had the lowest performance. All models provided similar performance for both amphibians and reptiles (Mann-Whitney tests for model-specific averaged cross-validated AUC, p-values >0.25). 

**Figure 2:**
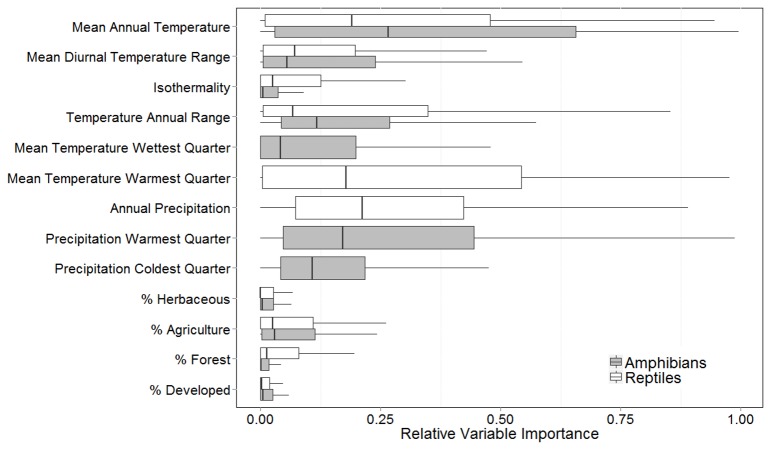
Box-and-whisker plot of relative importance of variables used to model amphibian and reptile distributions; computation of relative variable importance is described in the *Methods* section.

The contribution of variables to predicting species distributions ranged widely across species and modeling techniques (Figure 2). Overall, the land cover variables had a lower contribution to predicting current ranges compared to bioclimatic variables; the percent agricultural lands within the 100 km^2^ grid cells ranked highest among the land cover variables (median relative importance = 0.03 for both taxa). The best predictors for reptile distributions were the annual precipitation (0.20), the mean temperature of the warmest quarter (0.18), and mean annual temperature (0.19). For amphibians, the best predictors were mean annual temperature (0.27), and the precipitation of the warmest quarter (0.17).

The distributions of both amphibians and reptiles based on consensus predictions showed a diverse response to climate change across all emission and dispersal scenarios (Table 1). Under the no-dispersal scenario, the majority of the species (31 species for A1B and 32 species for A2A and B2A) lose suitable climate space by 2020s (i.e., ‘loser’ species) (median loss = 45.1% across all emission scenarios), with *Rana arvalis* being most affected (>90% reduction under all emission scenarios; Table 1). *Testudo hermanni* is predicted to completely lose climate space under the A2A and B2A scenarios. The 2050s time horizon shows a further reduction of suitable climate space for most species (median loss = 58.2%), and *Anguis fragilis*, *R. arvalis* and *R. lessonae* are predicted to completely lose suitable climate space. 

**Table 1:** Current range extent (number of occupied 10 × 10 km grid cells) and percent range change under future climate conditions (2020s and 2050s time horizons, emission scenarios A1B, A2A, and B2A) and dispersal assumptions, predicted using ensemble species distribution models; negative values denote range contractions (LimD = limited dispersal; NoD = no dispersal).

**Species**	**Current range**	**A1B2020s**		**A2A2020s**		**B2A20202s**		**A1B2050s**		**A2A2050s**		**B2A2050s**	
		**NoD**	**LimD**	**NoD**	**LimD**	**NoD**	**LimD**	**NoD**	**LimD**	**NoD**	**LimD**	**NoD**	**LimD**
**AMPHIBIANS**													
*Salamandra salamandra*	1190	-36.5	-36.5	-48.2	-48.2	-40.8	-40.3	-36.8	-36.7	-32.8	-32.3	-25.5	-24.0
*Triturus alpestris*	737	-19.5	-19.0	-25.6	-25.4	-28.5	-28.5	-30.5	-30.4	-35.1	-35.1	-35.3	-35.3
*Triturus cristatus*	1448	-44.5	-40.7	-41.6	-37.1	-60.2	-56.2	-84.6	-78.7	-79.6	-74.6	-84.5	-81.4
*Triturus dobrogicus*	244	-80.3	-80.3	-82.0	-82.0	-83.6	-83.6	-84.4	-84.4	-87.3	-87.3	-86.5	-86.5
*Triturus montandoni*	498	-28.1	-26.9	-17.1	-14.9	-39.4	-36.1	-61.4	-54.8	-53.6	-36.7	-47.0	-25.9
*Triturus vulgaris*	1425	-45.1	-40.1	-56.6	-54.7	-77.0	-74.9	-94.3	-93.0	-93.7	-92.8	-98.2	-98.0
*Bombina bombina*	1184	-19.6	-12.1	-20.7	-14.9	-26.4	-19.8	-27.9	-18.8	-26.4	-11.7	-25.7	-7.9
*Bombina variegata*	1392	-37.1	-37.1	-44.7	-44.7	-60.3	-60.3	-82.8	-82.8	-83.3	-83.3	-84.7	-84.7
*Pelobates fuscus*	641	-32.4	-26.1	-32.6	-27.0	-39.9	-36.2	-41.2	-32.4	-42.3	-34.9	-42.9	-31.5
*Pelobates syriacus*	119	0.0	63.0	-0.8	43.7	0.0	64.7	0.0	222.7	0.0	222.7	0.0	222.7
*Bufo bufo*	1342	-54.5	-52.8	-71.2	-69.7	-78.1	-77.5	-90.5	-90.3	-94.2	-94.1	-92.1	-91.9
*Bufo viridis*	1564	-30.2	-13.5	-50.6	-40.3	-39.8	-20.5	-44.8	-17.7	-41.1	-5.9	-22.1	25.8
*Hyla arborea*	1454	-39.8	-24.4	-46.4	-33.1	-66.2	-57.8	-71.7	-62.6	-67.1	-55.9	-46.1	-27.2
*Rana arvalis*	465	-94.2	-94.0	-98.5	-98.5	-99.6	-99.6	-100.0	-100.0	-100.0	-100.0	-100.0	-99.8
*Rana lessonae*	235	-85.5	-85.5	-80.9	-80.9	-98.7	-98.7	-99.6	-99.6	-99.6	-99.6	-100.0	-100.0
*Rana temporaria*	1269	-56.7	-56.7	-60.8	-60.8	-76.5	-76.5	-90.2	-90.2	-92.6	-92.6	-93.2	-93.2
**REPTILES**													
*Emys orbicularis*	801	-46.7	-34.2	-58.2	-48.4	-56.6	-44.7	-67.4	-51.2	-68.7	-57.3	-66.2	-57.4
*Testudo graeca*	194	0.0	43.3	-2.1	39.2	0.0	45.4	0.0	134.5	0.0	105.2	0.0	85.6
*Testudo hermanni*	111	-34.2	-14.4	-100.0	-100.0	-100.0	-100.0	-90.1	-89.2	-100.0	-100.0	-100.0	-100.0
*Anguis fragilis*	1167	-66.5	-66.5	-75.1	-75.1	-77.6	-77.6	-100.0	-100.0	-100.0	-100.0	-99.9	-99.9
*Eremias arguta*	59	0.0	27.1	0.0	28.8	0.0	28.8	0.0	127.1	0.0	105.1	0.0	64.4
*Lacerta agilis*	1643	-57.3	-57.2	-53.7	-52.6	-66.6	-65.2	-91.2	-91.1	-87.9	-87.3	-84.5	-83.8
*Lacerta praticola*	160	-16.9	23.8	-52.5	-40.0	-57.5	-45.6	-18.1	92.5	-28.8	63.8	-28.1	65.0
*Lacerta trilineata*	170	0.0	35.3	0.0	34.7	0.0	35.3	0.0	89.4	0.0	90.0	0.0	91.8
*Lacerta viridis*	1352	-12.6	0.1	-26.4	-13.5	-26.4	-13.6	-18.2	12.4	-25.6	2.3	-28.5	-5.5
*Podarcis muralis*	714	-50.1	-44.3	-94.5	-94.5	-89.5	-89.5	-81.6	-79.3	-97.9	-97.9	-95.0	-95.0
*Podarcis taurica*	516	0.0	28.1	0.0	28.7	0.0	29.1	0.0	85.3	0.0	86.2	0.0	85.5
*Lacerta vivipara*	803	-41.3	-41.3	-33.6	-32.4	-39.4	-38.7	-68.1	-68.1	-58.2	-58.2	-53.2	-53.2
*Ablepharus kitaibelii*	173	-36.4	-15.6	-45.7	-37.0	-50.9	-41.6	-36.4	16.8	-45.7	-7.5	-50.9	-13.9
*Coronella austriaca*	822	-73.6	-73.4	-89.4	-89.4	-90.6	-90.6	-99.5	-99.5	-99.9	-99.9	-99.4	-99.4
*Elaphe longissima*	762	-74.9	-73.6	-95.1	-94.8	-92.7	-92.1	-95.0	-85.7	-97.1	-95.7	-97.2	-96.2
*Coluber caspius*	207	-9.7	11.1	-18.4	-2.4	-18.8	-1.4	-21.2	63.8	-21.3	40.6	-21.3	38.2
*Elaphe quatuorlineata*	83	0.0	109.6	0.0	122.9	0.0	122.9	0.0	373.5	0.0	301.2	0.0	233.7
*Natrix tessellata*	848	-72.9	-65.4	-87.1	-79.1	-89.2	-82.7	-86.5	-80.1	-89.5	-87.9	-90.3	-88.6
*Vipera ammodytes*	289	-27.3	3.5	-64.0	-58.1	-64.7	-55.4	-33.5	88.9	-55.4	-15.6	-56.7	-32.9
*Vipera berus*	992	-52.3	-52.0	-53.8	-53.2	-61.6	-61.5	-88.0	-88.0	-83.6	-83.6	-78.4	-78.4
*Vipera ursinii*	49	-32.7	-20.4	-32.7	-20.4	-34.7	-24.5	-34.7	-22.4	-34.7	-20.4	-34.7	-20.4

Under the limited dispersal scenario, the median range loss is lower for both 2020s (40.1% across all emission scenarios), and 2050s (49.4%), but the magnitude and direction of change varies by species and emission scenario (Table 1). Among ’winner’ species, one amphibian (*Pelobates syriacus*) and eight reptile species (*Coluber caspius, Elaphe quatuorlineata, Eremias arguta, Lacerta praticola* [A1B only], *L. trilineata, Podarcis taurica, Testudo graeca*, and *Vipera ammodytes* [A1B only]) are predicted to gain suitable climate space for both 2020s and 2050s. Despite allowing for potential dispersal, severe reductions in the number of cells occupied (>90%) are predicted for some 2050s emission scenarios for *Bufo bufo, Triturus vulgaris, Coronella austriaca, Elaphe longissima*, and *Podarcis muralis* (Table 1). In addition, predictions of complete loss of suitable climate space are consistent with the no-dispersal scenario for both 2020s and 2050s. 

For both dispersal scenarios, range loses were highest under emission scenario B2A for 2020s, and A1B for 2050s (Table 1), but within each time horizon the differences there were not significant (asymptotic 3-sample permutation test; p-values >0.3). Across all emission scenarios, reptiles are predicted to lose suitable climate space at a slower pace compared to amphibians (Table 1), with the highest differences manifesting under the 2050s limited dispersal scenario (asymptotic 2-sample permutation test; p-value = 0.042). 

### Gap species

**Figure 3:**
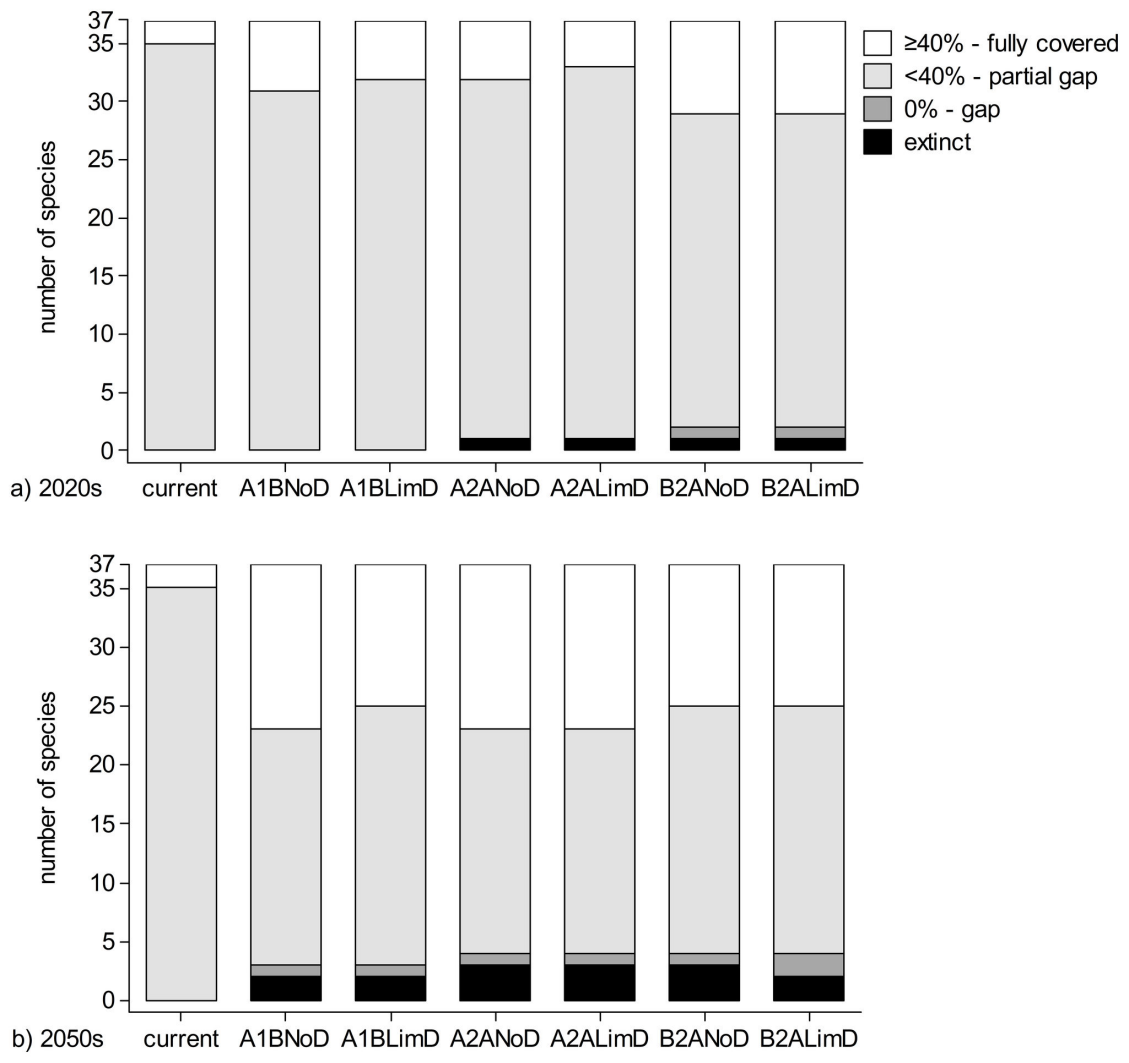
Representation of amphibian and reptile species in Romanian Natura 2000 sites under current and future climate conditions for limited- and no-dispersal scenarios (shown as percentage of planning units of species ranges in Sites of Community Importance).

Under current conditions all 37 reptile and amphibian species are represented at some degree in SCIs. Of these, 35 species (21 reptiles and 14 amphibians) are gap species (<40% of their current distribution in SCIs; Figure 3, Table S3, Table S4). Among the gap species, 12 species have <20% of their distribution in protected areas, with *Rana arvalis* (8.67%; species at its southern distribution limit) and *Bombina bombina* (10.23%; lowland species). The only two species that reach the 40% target are *Eremias arguta* (81.35%) and *Vipera ursinii* (62.5%); both species have very small and fragmented ranges overlapping with Natura 2000 sites.

For the 2020 and 2050 time horizons, the number of species that meet the 40% target increases (target met for 4 - 14 species depending on dispersal, time horizon and emission scenario; Figure 3, Table S3, Table S4). Concomitantly, the number of species that are marginally represented or are completely absent from SCIs increases. For example, *Testudo hermanni* completely loses suitable climate space under several emission scenarios, and *Rana arvalis* becomes either marginally protected or not represented in SCIs for all 2020 scenarios. Species such as *Triturus montandoni*, *Rana lessonae*, *Eremias arguta* and *Vipera ursinii* are predicted to be 100% represented in the current SCIs. In 2050, the number of marginally represented species increases, with *Coronella austriaca*, *Anguis fragilis*, *Testudo hermanni*, *Rana lessonae*, and *Triturus vulgaris* being most impacted.

###  Irreplaceability analysis

#### Current conditions

**Figure 4:**
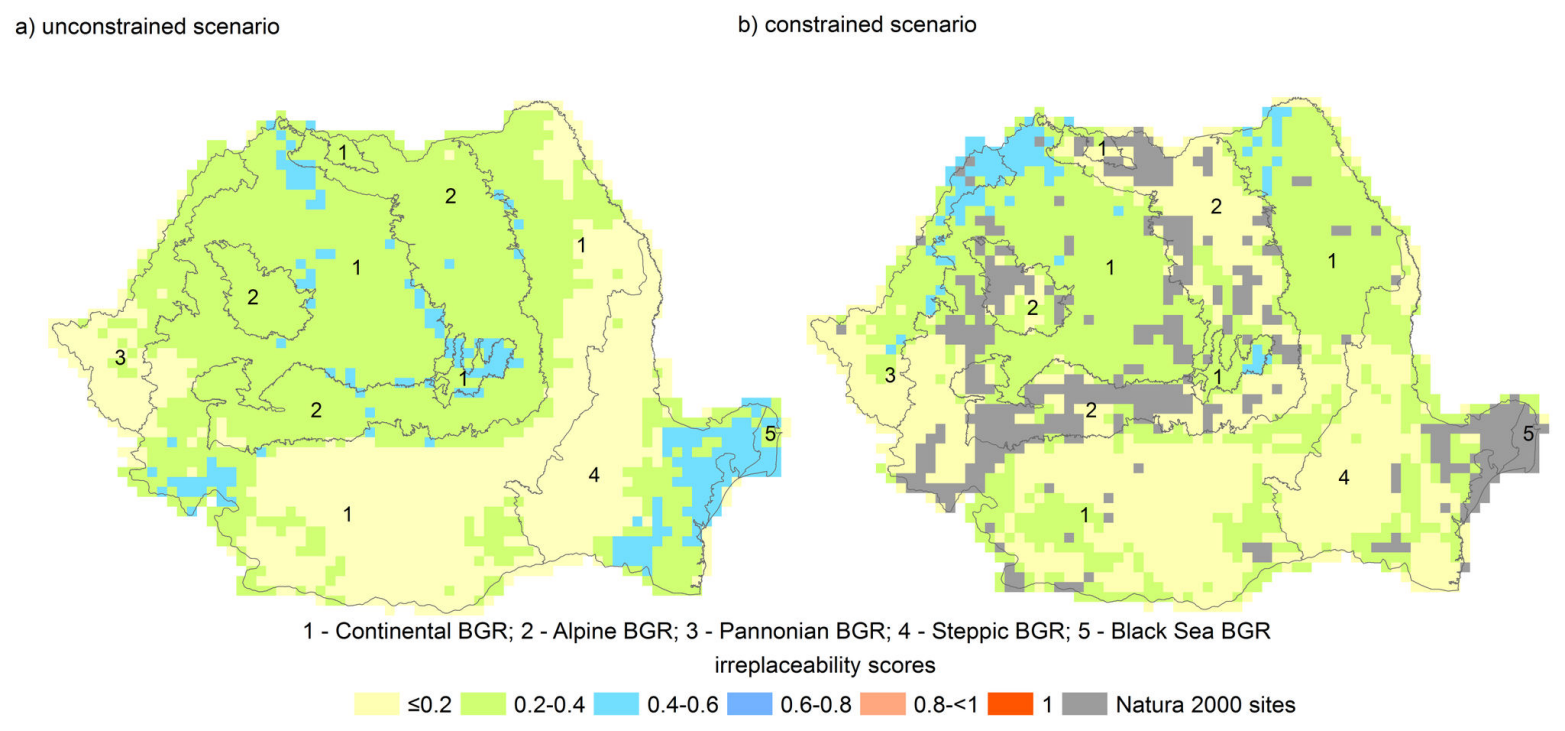
Planning unit (PU) irreplaceability scores under (a) unconstrained PU availability scenario (all PUs available for selection), and (b) constrained PU availability scenario (all currently protected PUs are given a score of 1). For (a), areas in blue represent priority areas for reptile and amphibian conservation regardless of their protection status. When all current SCIs are considered highly irreplaceable (b), northwest Romania becomes a conservation priority.

Currently, 92% of PUs (unconstrained availability) and 96% of PUs (constrained availability) have low and very low irreplaceability values (≤0.4). The remaining PUs under each scenario have moderate irreplaceability scores (0.4 - 0.6). Under the unconstrained availability scenario, 51% of PUs with moderate irreplaceability (N = 192) occur in SE Romania (Steppic and Black Sea BGRs), and 39% in SW and W Romania (Continental BGR), as well as the Alpine BGR (Figure 4), suggesting that conservation actions should focus on these regions regardless of their protection status. When the existing SCIs are considered irreplaceable, additional priority areas that currently do not benefit of protection occur mostly in NW Romania in two BGRs: Continental (N = 50 PUs), and Pannonian (N = 31 PUs; Figure 4).

#### Future conditions

**Figure 5:**
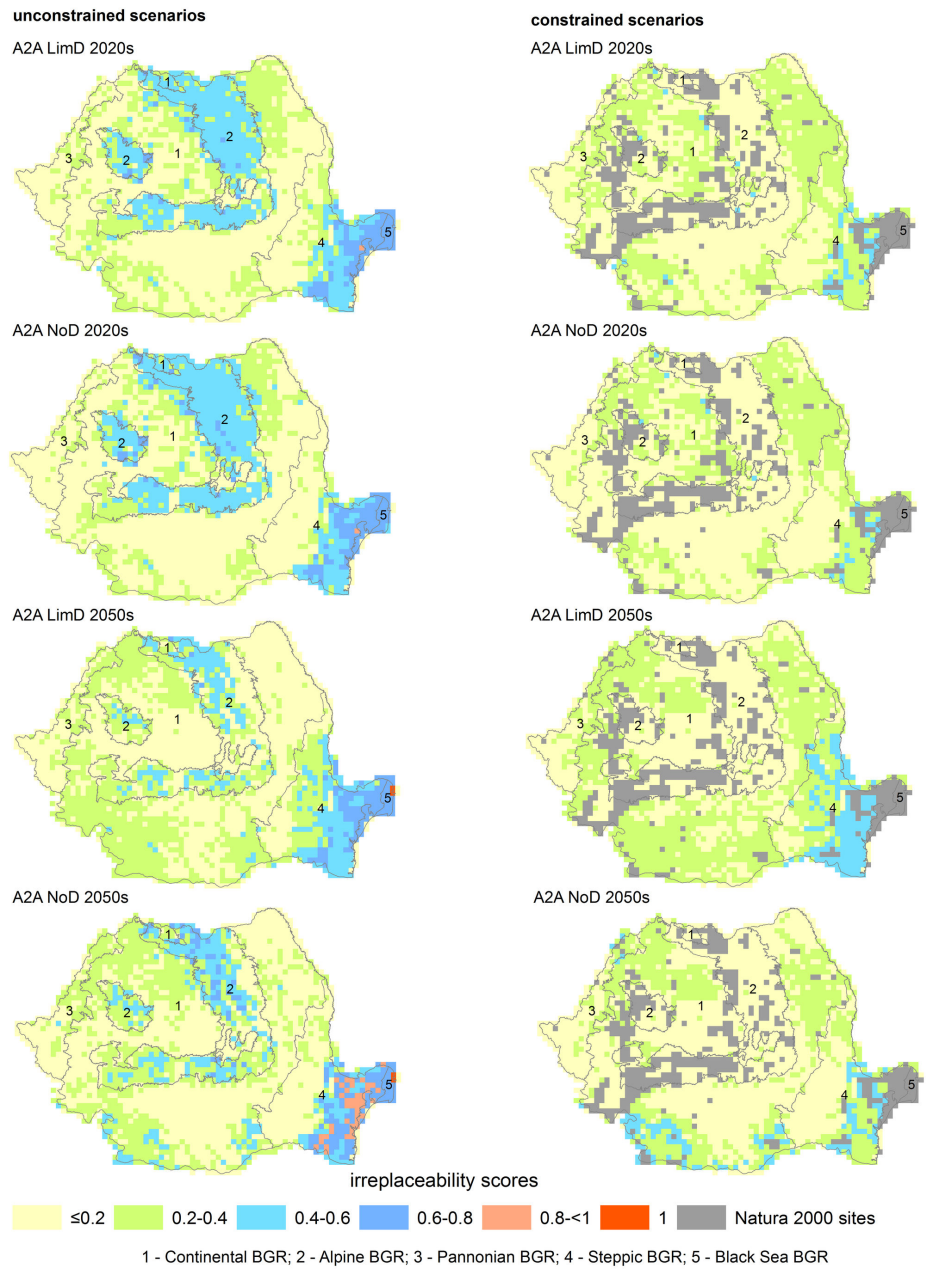
Example future irreplaceability scores (emission scenario A2A) under constrained (left panels) and unconstrained (right panels) planning unit availability for the 2020s and 2050s time horizons and three emission scenarios, under limited (LimD) and no dispersal (NoD) assumptions.

Under the unconstrained irreplaceability scenario, high and very high irreplaceability PUs (score >0.6) are predicted under all emission and dispersal scenarios (Figure S1). The Steppic-Black Sea, and Alpine BGRs have the highest irreplaceability values, regardless of emission and dispersal scenarios, with up to 154 PUs (6%) in categories high and very high in 2020s (B2A, no-dispersal), and up to 206 PUs (8%) in 2050s (A1A, no-dispersal) (Figure 5, Figure S2).

When existing SCIs are considered irreplaceable (constrained availability scenario), >95% of PUs have moderate and lower irreplaceability scores (<0.6) under all emission and dispersal scenarios (Figure S1). Similar to the current conditions, the Steppic-Black Sea BGRs have the highest priority highlighting the need to complementary protection in SE Romania. In addition, moderately valuable areas for herpetofauna conservation are predicted in SW and W Romania (e.g., B2A, no-dispersal) (Figure 5, Figure S2).

### Efficacy of Natura 2000

Natura 2000 sites have higher irreplaceability scores compared to a set of randomly sampled PUs under both the current climate and future climates for all emission and dispersal scenarios, suggesting that the current protected areas network is critical for achieving conservation targets for Romanian reptiles and amphibians (Table S5). However, we found a weak spatial association between protected areas (SCIs) and irreplaceability hotspots (φ <0.33) under both current and future conditions (Table 2). Current conditions had the weakest association, with 45.4% of SCIs overlapping irreplaceability hotspots, and φ = 0.14. In 2020s, the strength of the association increases up to 0.32 and 57.8% overlap (A2A, no-dispersal). In 2050s the spatial overlap recorded values similar to 2020s scenario (φ <0.33 and 46.59% overlap (A2A, no-dispersal, Table 2).

**Table 2:** Strength of association (coefficient of correlation *ϕ*) and percent overlap between hot-spots of irreplaceability and Natura 2000 planning units under current and future climate conditions (2020s and 2050, three emission scenarios) under limited dispersal (LimD) and no dispersal (NoD) assumptions.

**Climate scenario**		***ϕ***	**χ^2^ value**	**p-value**	**% overlap**
Current		0.14	49.94	<0.001	45.37
A1B2020s	NoD	0.32	260.92	<0.001	57.80
	LimD	0.31	244.86	<0.001	57.07
A2A2020s	NoD	0.28	199.76	<0.001	52.20
	LimD	0.28	199.76	<0.001	51.71
B2A2020s	NoD	0.30	229.32	<0.001	50.73
	LimD	0.28	199.76	<0.001	49.76
A1B2050s	NoD	0.33	277.48	<0.001	46.59
	LimD	0.23	134.79	<0.001	39.02
A2A2050s	NoD	0.31	244.86	<0.001	44.88
	LimD	0.23	134.79	<0.001	37.07
B2A2050s	NoD	0.32	260.92	<0.001	45.37
	LimD	0.29	214.29	<0.001	42.20

## Discussion

Our study showed that the forecasted climate changes will trigger important shifts of species ranges, with most species suffering range contractions (‘loser‘ species), while a few will benefit (‘winner’ species). Under current conditions, the existing network of SCIs does not perform well for conserving the reptile and amphibian fauna, and we identified new priority areas for conservation. In contrast to other studies (e.g., [46]), we found that under future climate conditions, the existing protected areas network is likely to increase the representation of the Romanian reptile and amphibian fauna, owing to the interaction between drastic range contractions, and range shifts towards currently protected areas. 

We used a biogeographic region approach to evaluate conservation priorities, and identified two core BGRs as critical to maintaining amphibian and reptile diversity: the Alpine and Steppic-Black Sea BGRs (Figure 4). These findings have important conservation implications. They suggest that despite known shortcomings of the Natura 2000 ecological network (e.g., uneven representation of European biogeographic regions, lack of connectivity, etc.; [16-18]), focusing conservation efforts on the existing SCIs (e.g., by improving their institutional capacity) is likely to improve the conservation status of herpetofauna in the future. We also recommend establishing new SCIs or incentivizing conservation actions in the priority areas outside protected lands would lead to better conservation status in the future.

### Predicted range changes – ‘loser’ and ‘winner’ species

There are differences in the responses of the two taxa, with amphibians unequivocally constricting their ranges during 2020s and 2050s, and reptiles showing mixed responses to climate change. Specifically, when considering limited dispersal, most amphibians are predicted to be ‘loser’ species, while among reptiles there are both ‘loser’ and ‘winner’ species (see Table 1). Such responses corroborate previous findings in Europe, which suggest that moisture and precipitation would become a limiting factor for amphibians, while climate warming might allow several reptile species to expand their ranges [15,46-48]. 

One of the interesting findings of our study was that *T. hermanni*, *A. fragilis*, *R. arvalis* and *R. lessonae* are predicted to completely lose climate space by 2050s under all emission scenarios (Table 1). This does not mean that species will completely disappear within a relatively short period of time (e.g., 2 generations for *T. hermanni*), but it is rather indicative of the fact that novel climates are likely to occur within these species’ current ranges. This raises an important ecological question: how will animals cope with novel climates which may have no analogues under current conditions [49,50]. The thermal tolerance breadth of ecotherms is greater in temperate latitudes of the Northern Hemisphere, compared to tropical latitudes [51,52]. This suggests that species whose distributions are highly influenced by temperature, such as reptiles, may persist in novel climates, despite our predictions of complete climate space loss. However, the responses to novel climates are unknown, and these findings suggest that niche-based species distribution models need to be revisited, by incorporating other sources of uncertainty, or rely on mechanistic relations between species and their environment [5]. In addition, there is limited evidence that anthropogenic climate change alone is responsible for species extinctions (e.g., through changes in temperature and moisture regimes which directly affect physiological tolerances), and that species interactions and food availability better correlate with extinction risk [53]. Another source of uncertainty is represented by the potential for evolutionary rescue (i.e., rapid adaptive responses to novel conditions), either through phenotypic plasticity [54,55] or genetic change [56]. For example, incorporating adaptation along with migration potential for predicting responses of 48 tree species in British Columbia to climate change, predicted the persistence of a higher number of tree populations compared to no-adaptation scenarios [7]. While the time horizon for our predictions is relatively narrow when considering evolutionary time scales, the short generation time and known plasticity of many amphibian and reptile species could potentially allow for evolutionary rescue. In addition, local adaptation would lead to different responses of populations across a species range, further adding to the uncertainty in predicting climate change impacts [57].

### Priority areas for herpetofauna conservation

Under the unconstrained irreplaceability scenario (i.e., regardless of existing protection status), both current and future conditions, the Alpine BGR (Carpathian Mountains) and the Steppic-Black Sea BGRs (SE Romania) have the highest priority for conservation of Romanian reptiles and amphibians (Figure 4, Figure 5, Figure S2). Agreement across multiple emission and dispersal scenarios and time horizons suggest that current and future conservation strategies should focus on these regions [20].

Our finding on the Alpine BRG as a high conservation priority area corroborates the results of continental-level studies [15,20], which also suggest that the Carpathians (especially Southern Carpathians) represent a top priority for European reptiles and amphibians. Such agreement between national and European level targets emphasizes the importance of the Romanian Alpine BGR to the conservation of European herpetofauna, while providing national-level priorities. The Carpathians host the majority of Romania’s forest ecosystems, and the resilience of the Alpine BGR to climate change is dependent on maintaining healthy forest ecosystems, and incorporating uncertainty into forest management strategies [58]. However, such issues gained little or no attention in Romania, and the most notable land use changes are taking place in the Carpathians. Deforestation represents the main driver of change, and rapid forest cover losses occurred in the post-socialist era through uncontrolled clear-cutting practices, which do not emulate the natural disturbance regimes [59]. Notably, such disturbances have been affecting, and continue to affect protected areas [60]. High rates of logging within and outside protected areas are the direct result of changes in land ownership in the post-socialist period [60], but also of weak regulatory, institutional, and enforcement potential of newly created protected areas [17]. Thus, in many parts of the Carpathians, forest cover has been reduced to fragments [61], which could affect the resilience of these ecosystems to climate change. Specifically for forest amphibians, which require both terrestrial and aquatic habitats during their complex lifecycles, there are no forest practices regulations that protect aquatic ecosystem integrity [62]. 

One of the findings of our study is identifying SE Romania (Dobrogea) as a priority area for herpetofauna conservation under both current and future climate scenarios (Figure 4, Figure 5, Figure S2). Due to its distinct location within three geographic barriers (Danube River at W and N, and Black Sea at E) Dobrogea is physically and functionally connected to the Balkan Peninsula, and has a unique herpetofauna (*T. graeca*, *Pelobates syriacus*, *Eremias arguta*, *Lacerta trilineata*, *Elaphe quatuorlineata* have the core range in SE Romania [22]), and high irreplaceability values. Abandonment of agricultural land is another common driver of land use change in post-socialist Romania [63], with large areas afforested and/or converted to natural vegetation [64]. Dobrogea is a region where cropland abandonment has been severe, and was concomitantly affected by droughts, soil erosion and desertification [65]. Decreased agricultural use and associated use of pesticides, and conversion to natural vegetation are likely to increase the likelihood that Dobrogea will remain a stronghold for Romanian herpetofauna in the future. In addition, focusing on abandoned croplands could reduce the likelihood for potential conflicts between conservation targets and economic interests of local communities.

However, partial mismatches between European level and national level predictions of top priority areas for conservation raise additional questions on how do national priorities play out in the overall vision for building a coherent Natura 2000 network [18,19,24].

### Efficacy of the Natura 2000 network for protecting amphibians and reptiles

The current Natura 2000 network does not meet European conservation targets for the majority of Romanian reptiles (N=19) and amphibians (N=16), yet many amphibians and reptiles are predicted to have higher levels of representation of under future emission scenarios. Shrinking distributions due to climate change can occasionally lead to increases in species representation in Natura 2000 network if the remaining ranges overlap with protected areas, potentially explaining why species such as *Triturus montandoni*, *Eremias arguta* or *Vipera ursinii* are predicted to become fully represented species in the future scenarios (Table S3, Table S4). At the same time, several other species (some of which are predicted to completely lose climate space) fail to be represented in the current ecological network (Table S3, Table S4). The findings on the performance of existing SCIs are not surprising, and they corroborate previous findings on the overall efficacy of the Romanian protected areas network. Using species inventories from existing SCIs [17], found that the majority of reptiles and amphibians were represented in at least one SCI, and that their overall protection level was inadequate. While representation in protected areas might be a good proxy for assessing a species conservation status, coverage alone does not warrant protection. In Romania, the total protected area increased from 4.1% to 22.7% of the national territory between 1990 and 2013, but it was not matched by an increase in efficacy of meeting conservation targets [17]. The result was an over-representation of undeveloped, yet potentially important resource extraction areas (mountains, floodplains, and Danube Delta), and under-representation of highly impacted areas, where immediate conservation measures are most needed [17,59]. As such, there are regional differences which highlight the under-representation of several BGRs in the current protected areas network. The Alpine BGR is best represented (47.8% of all Romanian SCIs), thus forest species and those associated with higher elevations benefit of the best protection. Species whose distributions are limited to Pannonian, Continental, and Steppic BGRs, located in NW, SW, and SE Romania, respectively tend to be under-represented.

The strength of association and spatial overlap between the Natura 2000 network and the irreplaceability hotspots increases under future climate projections, compared to current conditions (Table 2). This suggests that existing SCIs are critical for achieving future conservation targets for Romanian herpetofauna. Because no prior consideration was given to spatial planning under climate change scenarios [19], this finding can be explained by the fact that the Alpine and Steppic-Black Sea BGRs contain the bulk of Natura 2000 sites, and represent climate refugia for many amphibian and reptile species. 

Despite higher representation of amphibians and reptiles in protected areas under future conditions, approximately 75% (2020s scenarios) and 50% (2050s) of the species are still gap species (Figure 3). Within the two under-represented BGRs (i.e., Pannonian and Continental), conservation of reptiles and amphibians could be strengthened by establishing new SCIs, but also by promoting conservation strategies that make the local people an integral part of the solution [66,67]. For example, traditionally-managed areas in Romania are already recognized as important landscapes for reptile and amphibian conservation (e.g., [62,68]). Thus, an important next step would be to evaluate the overlap between these landscapes and future irreplaceability hotspots or distributions of high priority amphibian and reptile species. 

## Supporting Information

Table S1 **Maximum annual dispersal distances of adult individuals recorded for Romanian reptiles and amphibians.**
(DOCX)

Table S2 **Evaluation of model predictive performance: average cross-validated Area Under the Curve (AUC) of the Receiver Operating Characteristic (ROC) for seven models used in ensemble modeling of amphibian and reptile distributions in Romania (ANN = artificial neural networks; CTA = classification tree analysis; GAM = generalized additive models; GBM = generalized boosted regression trees; MARS = multivariate adaptive regression splines; FDA = flexible discriminant analysis; RF = random forests).**
(DOCX)

Table S3 **Percent conservation target met by amphibians and reptiles in Romanian Natura 2000 sites under current and under future climate conditions (2020s and 2050s time horizons, emission scenarios A1B, A2A, and B2A) and dispersal assumptions (LimD = limited dispersal; NoD = no dispersal); no-value cells represent species that are predicted to completely lose climate space.**
(DOCX)

Table S4 **Number of protected 10 ×10 km grid cells where amphibian and reptile species meet the 40% conservation target under current and under future climate conditions (2020s and 2050s time horizons, emission scenarios A1B, A2A, and B2A) and dispersal assumptions (LimD = limited dispersal; NoD = no dispersal); no-value cells represent species that are predicted to completely lose climate space.**
(DOCX)

Table S5 **Randomisations tests of differences between mean irreplaceability scores of Natura 2000 planning units and mean irreplaceability scores of randomly selected planning units under current and future climate conditions (2020s and 2050, three emission scenarios) under limited dispersal (LimD) and no dispersal (NoD) assumptions.**
(DOCX)

Figure S1 **Irreplaceability scores of planning units (10 ×10 km grid cells) for 2020s (a) and (b) and 2050s (c) and (d) under limited-dispersal (LimD) and no-dispersal (NoD) assumptions.**
(DOCX)

Figure S2 **Future irreplaceability scores for emission scenarios A1B (a) and B2A (b) under constrained (left panels) and unconstrained (right panels) planning unit availability for the 2020s and 2050s time horizons and three emission scenarios, under limited (LimD) and no dispersal (NoD) assumptions.**
(DOCX)
