# Correction: Moving into Protected Areas? Setting Conservation Priorities for Romanian Reptiles and Amphibians at Risk from Climate Change

**DOI:** 10.1371/annotation/cba407cc-1da1-4b23-ab8a-fc4b757a04ea

**Published:** 2014-01-06

**Authors:** Viorel D. Popescu, Laurenţiu Rozylowicz, Dan Cogălniceanu, Iulian Mihăiţă Niculae, Adina Livia Cucu

A grant from the Romanian National Authority for Scientific Research to the third author was incorrectly omitted from the Funding Statement. The Funding Statement should read: "This work was supported by a grant of the Romanian National Authority for Scientific Research, CNCS – UEFISCDI (http://uefiscdi.gov.ro), project number PN-II-RU-TE-2011-3-0183. DC was supported by a grant of the Romanian National Authority for Scientific Research, CNCS – UEFISCDI, project number PN-II-ID-PCE-2011-3-0173. VDP was partly supported by a David H. Smith Conservation Research Fellowship from the Society for Conservation Biology (www.smithfellows.org). The funders had no role in study design, data collection and analysis, decision to publish, or preparation of the manuscript."

In addition, the citation for Reference 22 is incorrect. The correct citation is: Cogălniceanu D, Rozylowicz L, Székely P, Samoilă C, Stănescu F et al. (2013) Diversity and distribution of reptiles in Romania. Zookeys 341: 49-76. doi: 10.3897/zookeys.341.5502. 

