# Supplementary material for: Moving into Protected Areas? Setting Conservation Priorities for Romanian Reptiles and Amphibians at Risk from Climate Change
Source: PLoS One. 2013 Nov 4;8(11):e79330. doi: 10.1371/journal.pone.0079330 (PMC3855577; doi:10.1371/journal.pone.0079330)
Supplement: Table S1 — Maximum annual dispersal distances of adult individuals recorded for Romanian reptiles and amphibians. (DOCX) [file pone.0079330.s001.docx]

*Moving into protected areas? Setting conservation priorities for Romanian reptiles and amphibians at risk from climate change*

Viorel D. Popescu, Laurenţiu Rozylowicz, Dan Cogălniceanu, Iulian Mihăiţă Niculae, Adina Livia Cucu

**Table S1**. Maximum annual dispersal distances of adult individuals recorded for Romanian reptiles and amphibians.

| **Species name^a^** | **Valid name^b^** | **Maximum annual dispersal (m)^c^** | **References** |
| --- | --- | --- | --- |
|  |  |  |  |
| **AMPHIBIANS** |  |  |  |
| *Salamandra salamandra* | *Salamandra salamandra* | 1000 | 3, 6, 7 |
| *Triturus alpestris* | *Ichthyosaura alpestris* | 1000 | 3, 6, 7 |
| *Triturus cristatus* | *Triturus cristatus* | 1000 | 3, 6, 7 |
| *Triturus dobrogicus* | *Triturus dobrogicus* | 1000 | 3, 6, 7 |
| *Triturus montandoni* | *Lissotriton montandoni* | 1000 | 3, 6, 7 |
| *Triturus vulgaris* | *Lissotriton vulgaris* | 1000 | 3, 6, 7 |
| *Bombina bombina* | *Bombina bombina* | 1500 | 2 |
| *Bombina variegata* | *Bombina variegata* | 1500 | 3, 6, 7 |
| *Pelobates fuscus* | *Pelobates fuscus* | 1000 | 3, 6, 7 |
| *Pelobates syriacus* | *Pelobates syriacus* | 1000 | 3, 6, 7 |
| *Bufo bufo* | *Bufo bufo* | 4000 | 3, 6, 7 |
| *Bufo viridis* | *Bufo viridis* | 1000 | 3, 6, 7 |
| *Hyla arborea* | *Hyla arborea* | 13000 | 3, 6, 7 |
| *Rana arvalis* | *Rana arvalis* | 8000 | 3, 6, 7 |
| *Rana lessonae* | *Pelophylax lessonae* | 15000 | 3, 6, 7 |
| *Rana temporaria* | *Rana temporaria* | 1000 | 3, 6, 7 |
|  |  |  |  |
| **REPTILES** |  |  |  |
| *Emys orbicularis* | *Emys orbicularis* | 5000 | 3, 6 |
| *Testudo graeca* | *Testudo graeca* | 2000 | 1 |
| *Testudo hermanni* | *Testudo hermanni* | 2000 | 5 |
| *Anguis fragilis* | *Anguis fragilis* | 1000 | 3, 6 |
| *Eremias arguta* | *Eremias arguta* | 1000 | 3, 6 |
| *Lacerta agilis* | *Lacerta agilis* | 1000 | 3, 6 |
| *Lacerta praticola* | *Darevskia praticola* | 1000 | 3, 6 |
| *Lacerta trilineata* | *Lacerta trilineata* | 1000 | 3, 6 |
| *Lacerta viridis* | *Lacerta viridis* | 1000 | 3, 6 |
| *Podarcis muralis* | *Podarcis muralis* | 1000 | 3, 6 |
| *Podarcis taurica* | *Podarcis tauricus* | 1000 | 3, 6 |
| *Lacerta vivipara* | *Zootoca vivipara* | 1000 | 3, 6 |
| *Ablepharus kitaibelii* | *Ablepharus kitaibelii* | 1000 | 3, 6 |
| *Coronella austriaca* | *Coronella austriaca* | 2000 | 3, 6 |
| *Elaphe longissima* | *Zamenis longissimus* | 2000 | 3, 6 |
| *Coluber caspius* | *Dolichophis caspius* | 2000 | 3, 6 |
| *Elaphe quatuorlineata* | *Elaphe sauromates* | 2000 | 3, 6 |
| *Natrix tessellata* | *Natrix tessellata* | 10000 | 3, 6 |
| *Vipera ammodytes* | *Vipera ammodytes* | 2000 | 3, 4, 6 |
| *Vipera berus* | *Vipera berus* | 2000 | 3, 4, 6 |
| *Vipera ursinii* | *Vipera ursinii* | 2000 | 3, 4, 6 |

^a^ – species names used by European Habitats Directive and related Romanian legislation

^b^ – after Speybroeck, J., Beukema. W. and P.A. Crochet. 2010. A tentative species list of the European herpetofauna (Amphibia and Reptilia) - an update. Zootaxa 2492: 1-27.

^c^ – References for maximum annual dispersal distances

1. Buskirk, J. R., Keller, C. and A.C. Andreu. 2001. *Testudo graeca* Linnaeus, 1758–Maurische Landschildkröte. Handbuch der Reptilien und Amphibien Europas, 3, 125-178.
2. Hartel, T. 2008. Movement activity in a *Bombina variegata* population from a deciduous forested landscape. North-Western Journal of Zoology 4: 79-90.
3. Henle, M.K., Dick, D., Harpke, A., Kühn, I., Schweiger, O. and J. Settele. 2010. Climate change impacts on European amphibians and reptiles. Biodiversity and climate change: Reports and guidance developed under the Bern Convention. Council of Europe, Strasbourg.
4. Joger, U. and N. Stümpel. 2005. Schlangen 3 - Vipern. Handbuch der Reptilien und Amphibien Europas., AULA-Verlag GmbH, Wiebelsheim, Germany.
5. Rozylowicz, L. and V. D. Popescu. 2013. Habitat selection and movement ecology of eastern Hermann’s tortoises in a rural Romanian landscape. European Journal of Wildlife Research 59: 47-55.
6. Russell, A., Bauer, A., and M. Johnson. 2005. Migration in amphibians and reptiles: An overview of patterns and orientation mechanisms in relation to life history strategies. Pages 151-203 *in* A. T. Elewa, editor. Migration of Organisms. Springer, Berlin Heidelberg.
7. Smith, A. M. and D. M. Green. 2005. Dispersal and the metapopulation paradigm in amphibian ecology and conservation: are all amphibian populations metapopulations? Ecography 28: 110-128.
