# Supplementary material for: Moving into Protected Areas? Setting Conservation Priorities for Romanian Reptiles and Amphibians at Risk from Climate Change
Source: PLoS One. 2013 Nov 4;8(11):e79330. doi: 10.1371/journal.pone.0079330 (PMC3855577; doi:10.1371/journal.pone.0079330)
Supplement: Table S2 — Evaluation of model predictive performance: average cross-validated Area Under the Curve (AUC) of the Receiver Operating Characteristic (ROC) for seven models used in ensemble modeling of amphibian and reptile distributions in Romania (ANN = artificial neural networks; CTA = classification tree analysis; GAM = generalized additive models; GBM = generalized boosted regression trees; MARS = multivariate adaptive regression splines; FDA = flexible discriminant analysis; RF = random forests). (DOCX) [file pone.0079330.s002.docx]

*Moving into protected areas? Setting conservation priorities for Romanian reptiles and amphibians at risk from climate change*

Viorel D. Popescu, Laurenţiu Rozylowicz, Dan Cogălniceanu, Iulian Mihăiţă Niculae, Adina Livia Cucu

**Table S2.** Evaluation of model predictive performance: average cross-validated Area Under the Curve (AUC) of the Receiver Operating Characteristic (ROC) for seven models used in ensemble modeling of amphibian and reptile distributions in Romania (ANN = artificial neural networks; CTA = classification tree analysis; GAM = generalized additive models; GBM = generalized boosted regression trees; MARS = multivariate adaptive regression splines; FDA = flexible discriminant analysis; RF = random forests).

| **Species** | **ANN** | **CTA** | **GAM** | **GBM** | **MARS** | **FDA** | **RF** |
| --- | --- | --- | --- | --- | --- | --- | --- |
| **AMPHIBIANS** |  |  |  |  |  |  |  |
| *Salamandra salamandra* | 0.954 | 0.928 | 0.970 | 0.964 | 0.957 | 0.961 | 0.969 |
| *Triturus alpestris* | 0.957 | 0.914 | 0.965 | 0.967 | 0.966 | 0.961 | 0.972 |
| *Triturus cristatus* | 0.918 | 0.893 | 0.922 | 0.938 | 0.913 | 0.905 | 0.953 |
| *Triturus dobrogicus* | 0.946 | 0.945 | 0.957 | 0.982 | 0.956 | 0.966 | 0.986 |
| *Triturus montandoni* | 0.979 | 0.972 | 0.988 | 0.983 | 0.986 | 0.975 | 0.989 |
| *Triturus vulgaris* | 0.909 | 0.882 | 0.906 | 0.923 | 0.916 | 0.899 | 0.952 |
| *Bombina bombina* | 0.915 | 0.870 | 0.904 | 0.928 | 0.908 | 0.894 | 0.955 |
| *Bombina variegata* | 0.986 | 0.956 | 0.989 | 0.991 | 0.979 | 0.974 | 0.991 |
| *Pelobates fuscus* | 0.848 | 0.769 | 0.880 | 0.901 | 0.876 | 0.882 | 0.916 |
| *Pelobates syriacus* | 0.983 | 0.912 | 0.977 | 0.992 | 0.932 | 0.986 | 0.994 |
| *Bufo bufo* | 0.940 | 0.901 | 0.941 | 0.954 | 0.949 | 0.946 | 0.974 |
| *Bufo viridis* | 0.846 | 0.803 | 0.829 | 0.866 | 0.840 | 0.833 | 0.915 |
| *Hyla arborea* | 0.877 | 0.810 | 0.864 | 0.889 | 0.851 | 0.874 | 0.927 |
| *Rana arvalis* | 0.929 | 0.834 | 0.935 | 0.942 | 0.944 | 0.952 | 0.968 |
| *Rana lessonae* | 0.823 | 0.800 | 0.876 | 0.855 | 0.818 | 0.795 | 0.849 |
| *Rana temporaria* | 0.986 | 0.983 | 0.993 | 0.994 | 0.992 | 0.978 | 0.997 |
|  |  |  |  |  |  |  |  |
| **REPTILES** |  |  |  |  |  |  |  |
| *Emys orbicularis* | 0.815 | 0.744 | 0.818 | 0.844 | 0.817 | 0.801 | 0.868 |
| *Testudo graeca* | 0.995 | 0.942 | 0.990 | 0.989 | 0.976 | 0.982 | 0.995 |
| *Testudo hermanni* | 0.977 | 0.888 | 0.982 | 0.981 | 0.989 | 0.989 | 0.988 |
| *Anguis fragilis* | 0.921 | 0.896 | 0.940 | 0.946 | 0.932 | 0.935 | 0.953 |
| *Eremias arguta* | 0.938 | 0.865 | 0.938 | 0.993 | 0.937 | 0.940 | 0.939 |
| *Lacerta agilis* | 0.920 | 0.852 | 0.904 | 0.911 | 0.906 | 0.898 | 0.938 |
| *Lacerta praticola* | 0.833 | 0.618 | 0.786 | 0.853 | 0.822 | 0.864 | 0.854 |
| *Lacerta trilineata* | 0.989 | 0.960 | 0.990 | 0.986 | 0.993 | 0.988 | 0.994 |
| *Lacerta virdis* | 0.872 | 0.836 | 0.840 | 0.888 | 0.866 | 0.846 | 0.934 |
| *Podarcis muralis* | 0.926 | 0.888 | 0.940 | 0.931 | 0.930 | 0.925 | 0.944 |
| *Podarcis taurica* | 0.962 | 0.935 | 0.983 | 0.981 | 0.979 | 0.968 | 0.989 |
| *Lacerta vivipara* | 0.964 | 0.930 | 0.971 | 0.977 | 0.958 | 0.960 | 0.981 |
| *Ablepharus kitaibelii* | 0.849 | 0.754 | 0.880 | 0.924 | 0.866 | 0.897 | 0.924 |
| *Coronella austriaca* | 0.803 | 0.830 | 0.871 | 0.890 | 0.846 | 0.851 | 0.901 |
| *Elaphe longissima* | 0.870 | 0.832 | 0.854 | 0.881 | 0.858 | 0.865 | 0.915 |
| *Coluber caspius* | 0.936 | 0.889 | 0.932 | 0.945 | 0.936 | 0.916 | 0.942 |
| *Elaphe quatuorlineata* | 0.987 | 0.938 | 0.985 | 0.985 | 0.910 | 0.980 | 0.992 |
| *Natrix tessellata* | 0.861 | 0.834 | 0.886 | 0.902 | 0.902 | 0.882 | 0.928 |
| *Vipera ammodytes* | 0.937 | 0.862 | 0.933 | 0.942 | 0.930 | 0.925 | 0.962 |
| *Vipera berus* | 0.908 | 0.888 | 0.924 | 0.938 | 0.923 | 0.920 | 0.951 |
| *Vipera ursinii* | 0.876 | 0.765 | 0.811 | 0.890 | 0.919 | 0.876 | 0.846 |
