# Supplementary material for: Moving into Protected Areas? Setting Conservation Priorities for Romanian Reptiles and Amphibians at Risk from Climate Change
Source: PLoS One. 2013 Nov 4;8(11):e79330. doi: 10.1371/journal.pone.0079330 (PMC3855577; doi:10.1371/journal.pone.0079330)
Supplement: Table S3 — Percent conservation target met by amphibians and reptiles in Romanian Natura 2000 sites under current and under future climate conditions (2020s and 2050s time horizons, emission scenarios A1B, A2A, and B2A) and dispersal assumptions (LimD = limited dispersal; NoD = no dispersal); no-value cells represent species that are predicted to completely lose climate space. (DOCX) [file pone.0079330.s003.docx]

*Moving into protected areas? Setting conservation priorities for Romanian reptiles and amphibians at risk from climate change*

Viorel D. Popescu, Laurenţiu Rozylowicz, Dan Cogălniceanu, Iulian Mihăiţă Niculae, Adina Livia Cucu

**Table S3.** Percent conservation target met by amphibians and reptiles in Romanian Natura 2000 sites under current and under future climate conditions (2020s and 2050s time horizons, emission scenarios A1B, A2A, and B2A) and dispersal assumptions (LimD = limited dispersal; NoD = no dispersal); no-value cells represent species that are predicted to completely lose climate space.

| **Species** | **Current** | **A1B2020s** | | **A1B205s0** | | **A2A2020s** | | **A2A2050s** | | **B2A2020s** | | **B2A2050s** | |
| --- | --- | --- | --- | --- | --- | --- | --- | --- | --- | --- | --- | --- | --- |
|  |  | **NoD** | **LimD** | **NoD** | **LimD** | **NoD** | **LimD** | **NoD** | **LimD** | **NoD** | **LimD** | **NoD** | **LimD** |
| **AMPHIBIANS** |  |  |  |  |  |  |  |  |  |  |  |  |  |
| *Salamandra salamandra* | 63.57 | 81.76 | 81.76 | 76.90 | 76.79 | 87.58 | 87.58 | 73.75 | 73.38 | 85.25 | 84.40 | 70.64 | 69.51 |
| *Triturus alpestris* | 83.33 | 95.89 | 96.09 | 97.03 | 96.84 | 96.30 | 95.94 | 100.0 | 100.0 | 96.63 | 96.63 | 100.00 | 100.00 |
| *Triturus cristatus* | 32.33 | 44.12 | 50.78 | 70.78 | 89.11 | 43.13 | 49.27 | 61.42 | 75.00 | 47.68 | 55.02 | 47.67 | 59.62 |
| *Triturus dobrogicus* | 66.60 | 100.00 | 100.0 | 100.00 | 100.00 | 100.00 | 100.00 | 100.00 | 100.0 | 100.0 | 100.00 | 100.00 | 100.00 |
| *Triturus montandoni* | 53.35 | 64.63 | 66.53 | 73.92 | 72.09 | 58.35 | 61.00 | 75.56 | 65.79 | 68.41 | 71.54 | 74.61 | 62.50 |
| *Triturus vulgaris* | 36.41 | 45.97 | 51.82 | 79.11 | 86.73 | 42.98 | 43.98 | 56.18 | 56.93 | 40.75 | 45.39 | 31.25 | 27.78 |
| *Bombina bombina* | 25.58 | 29.43 | 26.89 | 31.29 | 29.12 | 29.47 | 28.19 | 32.23 | 28.29 | 31.43 | 29.93 | 31.65 | 28.76 |
| *Bombina variegata* | 55.31 | 75.03 | 75.03 | 100.00 | 100.00 | 75.43 | 75.43 | 100.00 | 100.0 | 87.50 | 87.50 | 100.00 | 100.00 |
| *Pelobates fuscus* | 39.76 | 54.78 | 50.53 | 52.81 | 47.09 | 55.62 | 51.30 | 59.26 | 53.74 | 60.21 | 57.27 | 55.25 | 48.28 |
| *Pelobates syriacus* | 92.44 | 92.44 | 73.45 | 92.44 | 57.94 | 93.22 | 80.41 | 92.44 | 57.94 | 92.44 | 77.81 | 92.44 | 57.94 |
| *Bufo bufo* | 56.96 | 89.32 | 89.23 | 100.00 | 100.00 | 96.20 | 95.00 | 100.00 | 100.0 | 100.0 | 100.00 | 100.00 | 100.00 |
| *Bufo viridis* | 37.48 | 41.67 | 39.52 | 44.07 | 45.08 | 45.60 | 45.77 | 45.93 | 48.45 | 41.76 | 48.17 | 41.35 | 47.88 |
| *Hyla arborea* | 36.72 | 43.45 | 41.74 | 58.79 | 53.74 | 44.06 | 40.93 | 53.72 | 49.13 | 54.17 | 55.65 | 47.14 | 47.70 |
| *Rana arvalis* | 21.67 | 27.78 | 26.79 | **-** | **-** | 35.71 | 35.71 | *-* | *-* | 0.00 | 0.00 | **-** | 0.00 |
| *Rana lessonae* | 41.49 | 100.00 | 100.0 | 0.00 | 0.00 | 72.22 | 72.22 | 100.00 | 0.00 | 83.33 | 83.33 | **-** | **-** |
| *Rana temporaria* | 59.78 | 94.10 | 94.10 | 100.00 | 100.00 | 92.35 | 92.35 | 100.00 | 100.00 | 100.00 | 100.00 | 100.00 | 100.00 |
|  |  |  |  |  |  |  |  |  |  |  |  |  |  |
| **REPTILES** |  |  |  |  |  |  |  |  |  |  |  |  |  |
| *Emys orbicularis* | 40.80 | 52.79 | 45.41 | 69.61 | 50.00 | 59.05 | 50.12 | 74.07 | 57.63 | 57.12 | 47.45 | 68.70 | 57.23 |
| *Testudo graeca* | 73.45 | 73.45 | 66.55 | 73.45 | 42.95 | 75.00 | 68.52 | 73.45 | 47.86 | 73.45 | 66.49 | 73.45 | 52.23 |
| *Testudo hermanni* | 78.83 | 100.00 | 100.00 | 100.00 | 100.00 | **-** | **-** | **-** | **-** | **-** | **-** | **-** | **-** |
| *Anguis fragilis* | 64.20 | 97.40 | 97.40 | **-** | **-** | 95.61 | 95.61 | **-** | **-** | 100.00 | 100.00 | 0.00 | 0.00 |
| *Eremias arguta* | 100.00 | 100.00 | 100.00 | 100.00 | 100.00 | 100.00 | 100.00 | 100.00 | 100.00 | 100.00 | 100.00 | 100.00 | 100.00 |
| *Lacerta agilis* | 53.73 | 80.81 | 80.82 | 100.00 | 100.00 | 71.38 | 71.10 | 100.00 | 100.00 | 92.42 | 90.87 | 100.00 | 100.00 |
| *Lacerta praticola* | 73.72 | 71.71 | 54.12 | 78.74 | 55.92 | 66.67 | 55.26 | 89.29 | 61.54 | 88.24 | 71.84 | 81.86 | 58.21 |
| *Lacerta trilineata* | 80.88 | 80.88 | 69.57 | 80.88 | 55.12 | 80.88 | 68.78 | 80.88 | 54.95 | 80.88 | 69.57 | 80.88 | 56.75 |
| *Lacerta virdis* | 35.61 | 33.96 | 30.78 | 26.88 | 21.29 | 26.33 | 23.68 | 23.33 | 18.36 | 26.64 | 24.15 | 22.06 | 18.62 |
| *Podarcis muralis* | 76.90 | 96.15 | 92.88 | 100.00 | 100.00 | 76.92 | 76.92 | 100.00 | 100.0 | 93.75 | 93.75 | 83.33 | 83.33 |
| *Podarcis taurica* | 39.53 | 39.53 | 37.63 | 39.53 | 34.85 | 39.53 | 38.61 | 39.53 | 36.24 | 39.53 | 38.87 | 39.53 | 35.64 |
| *Lacerta vivipara* | 71.29 | 96.98 | 96.98 | 100.00 | 100.00 | 90.38 | 89.15 | 100.00 | 100.00 | 94.14 | 93.69 | 100.00 | 100.00 |
| *Ablepharus kitaibelii* | 59.25 | 63.64 | 63.36 | 70.45 | 67.73 | 71.81 | 73.39 | 71.81 | 81.25 | 76.47 | 84.16 | 70.59 | 82.21 |
| *Coronella austriaca* | 48.74 | 47.33 | 48.08 | 100.00 | 100.00 | 41.14 | 41.14 | 0.00 | 100.0 | 38.19 | 38.19 | 50.00 | 50.00 |
| *Elaphe longissima* | 60.30 | 96.05 | 95.00 | 100.00 | 46.73 | 100.00 | 93.75 | 100.00 | 100.0 | 100.00 | 100.00 | 100.00 | 100.00 |
| *Coluber caspius* | 79.71 | 74.87 | 70.65 | 58.28 | 32.54 | 63.61 | 53.22 | 59.82 | 43.10 | 62.50 | 61.27 | 59.82 | 47.37 |
| *Elaphe quatuorlineata* | 57.23 | 57.23 | 51.72 | 57.23 | 29.34 | 57.23 | 51.35 | 57.23 | 36.90 | 57.23 | 52.70 | 57.23 | 38.04 |
| *Natrix tessellata* | 56.33 | 100.00 | 94.50 | 100.00 | 99.11 | 100.00 | 100.00 | 100.00 | 100.00 | 100.00 | 100.00 | 100.00 | 100.00 |
| *Vipera ammodytes* | 87.37 | 95.24 | 95.96 | 84.64 | 61.00 | 64.29 | 65.57 | 81.40 | 71.28 | 71.78 | 80.08 | 80.00 | 83.33 |
| *Vipera berus* | 68.25 | 92.08 | 92.02 | 100.00 | 100.00 | 93.82 | 93.14 | 100.00 | 100.00 | 98.00 | 97.74 | 100.00 | 100.00 |
| *Vipera ursinii* | 100.00 | 100.00 | 100.00 | 100.00 | 100.00 | 100.00 | 100.00 | 100.00 | 100.00 | 100.00 | 100.00 | 100.00 | 100.00 |
