# Supplementary material for: Moving into Protected Areas? Setting Conservation Priorities for Romanian Reptiles and Amphibians at Risk from Climate Change
Source: PLoS One. 2013 Nov 4;8(11):e79330. doi: 10.1371/journal.pone.0079330 (PMC3855577; doi:10.1371/journal.pone.0079330)
Supplement: Table S4 — Number of protected 10 ×10 km grid cells where amphibian and reptile species meet the 40% conservation target under current and under future climate conditions (2020s and 2050s time horizons, emission scenarios A1B, A2A, and B2A) and dispersal assumptions (LimD = limited dispersal; NoD = no dispersal); no-value cells represent species that are predicted to completely lose climate space. (DOCX) [file pone.0079330.s004.docx]

*Moving into protected areas? Setting conservation priorities for Romanian reptiles and amphibians at risk from climate change*

Viorel D. Popescu, Laurenţiu Rozylowicz, Dan Cogălniceanu, Iulian Mihăiţă Niculae, Adina Livia Cucu

**Table S4.** Number of protected 10 ×10 km grid cells where amphibian and reptile species meet the 40% conservation target under current and under future climate conditions (2020s and 2050s time horizons, emission scenarios A1B, A2A, and B2A) and dispersal assumptions (LimD = limited dispersal; NoD = no dispersal); no-value cells represent species that are predicted to completely lose climate space.

| **Species** | **Curent** | **A1B2020s** | | **A1B2050s** | | **A2A2020s** | | **A2A2050s** | | **B2A2020s** | | **B2A2050s** | |
| --- | --- | --- | --- | --- | --- | --- | --- | --- | --- | --- | --- | --- | --- |
|  |  | **NoD** | **LimD** | **NoD** | **LimD** | **NoD** | **LimD** | **NoD** | **LimD** | **NoD** | **LimD** | **NoD** | **LimD** |
| **AMPHIBIANS** |  |  |  |  |  |  |  |  |  |  |  |  |  |
| *Salamandra salamandra* | 296 | 242 | 242 | 227 | 227 | 213 | 213 | 231 | 231 | 237 | 237 | 243 | 243 |
| *Triturus alpestris* | 242 | 224 | 226 | 196 | 196 | 208 | 208 | 188 | 188 | 201 | 201 | 188 | 188 |
| *Triturus cristatus* | 183 | 138 | 170 | 62 | 108 | 142 | 175 | 71 | 108 | 107 | 136 | 41 | 62 |
| *Triturus dobrogicus* | 65 | 19 | 19 | 15 | 15 | 18 | 18 | 12 | 12 | 16 | 16 | 13 | 13 |
| *Triturus montandoni* | 105 | 91 | 95 | 55 | 62 | 95 | 102 | 68 | 80 | 81 | 89 | 77 | 89 |
| *Triturus vulgaris* | 203 | 139 | 171 | 25 | 34 | 104 | 111 | 20 | 23 | 52 | 63 | 3 | 3 |
| *Bombina bombina* | 119 | 111 | 111 | 106 | 111 | 110 | 113 | 111 | 117 | 109 | 113 | 110 | 124 |
| *Bombina variegata* | 300 | 256 | 256 | 94 | 94 | 226 | 226 | 92 | 92 | 189 | 189 | 83 | 83 |
| *Pelobates fuscus* | 101 | 94 | 95 | 79 | 81 | 95 | 95 | 87 | 89 | 92 | 93 | 80 | 84 |
| *Pelobates syriacus* | 44 | 44 | 57 | 44 | 89 | 44 | 55 | 44 | 89 | 44 | 61 | 44 | 89 |
| *Bufo bufo* | 298 | 214 | 222 | 50 | 51 | 147 | 152 | 31 | 32 | 116 | 119 | 42 | 43 |
| *Bufo viridis* | 230 | 180 | 212 | 150 | 229 | 139 | 169 | 167 | 282 | 156 | 237 | 198 | 370 |
| *Hyla arborea* | 209 | 150 | 181 | 95 | 115 | 135 | 157 | 101 | 124 | 104 | 134 | 145 | 199 |
| *Rana arvalis* | 39 | 3 | 3 | **-** | **-** | 1 | 1 | **-** | **-** | 0 | 0 | **-** | 0 |
| *Rana lessonae* | 39 | 14 | 14 | 0 | 0 | 13 | 13 | 0 | 0 | 1 | 1 | **-** | **-** |
| *Rana temporaria* | 297 | 204 | 204 | 48 | 48 | 181 | 181 | 37 | 37 | 118 | 118 | 34 | 34 |
|  |  |  |  |  |  |  |  |  |  |  |  |  |  |
| **REPTILES** |  |  |  |  |  |  |  |  |  |  |  |  |  |
| *Emys orbicularis* | 126 | 87 | 93 | 71 | 77 | 77 | 81 | 72 | 77 | 77 | 82 | 72 | 76 |
| *Testudo graeca* | 57 | 57 | 74 | 57 | 78 | 57 | 74 | 57 | 76 | 57 | 75 | 57 | 75 |
| *Testudo hermanni* | 35 | 29 | 38 | 4 | 5 | **-** | **-** | **-** | **-** | **-** | **-** | **-** | **-** |
| *Anguis fragilis* | 292 | 150 | 150 | **-** | **-** | 109 | 109 | **-** | **-** | 102 | 102 | 0 | 0 |
| *Eremias arguta* | 24 | 24 | 30 | 24 | 54 | 24 | 30 | 24 | 48 | 24 | 30 | 24 | 39 |
| *Lacerta agilis* | 346 | 224 | 225 | 57 | 58 | 215 | 219 | 79 | 83 | 200 | 205 | 100 | 105 |
| *Lacerta praticola* | 46 | 37 | 42 | 40 | 68 | 20 | 21 | 40 | 64 | 24 | 25 | 37 | 61 |
| *Lacerta trilineata* | 55 | 55 | 64 | 55 | 71 | 55 | 63 | 55 | 71 | 55 | 64 | 55 | 74 |
| *Lacerta virdis* | 188 | 158 | 164 | 116 | 127 | 103 | 109 | 92 | 100 | 104 | 111 | 84 | 94 |
| *Podarcis muralis* | 215 | 135 | 146 | 50 | 57 | 12 | 12 | 6 | 6 | 27 | 27 | 12 | 12 |
| *Podarcis taurica* | 80 | 80 | 98 | 80 | 132 | 80 | 101 | 80 | 138 | 80 | 102 | 80 | 135 |
| *Lacerta vivipara* | 225 | 180 | 180 | 100 | 100 | 188 | 189 | 132 | 131 | 180 | 181 | 148 | 148 |
| *Ablepharus kitaibelii* | 41 | 28 | 37 | 31 | 55 | 27 | 32 | 27 | 52 | 26 | 34 | 24 | 49 |
| *Coronella austriaca* | 155 | 39 | 40 | 2 | 2 | 13 | 13 | 0 | 0 | 11 | 11 | 1 | 1 |
| *Elaphe longissima* | 178 | 73 | 76 | 14 | 20 | 15 | 15 | 9 | 13 | 22 | 24 | 8 | 12 |
| *Coluber caspius* | 66 | 56 | 65 | 38 | 44 | 43 | 43 | 39 | 50 | 42 | 50 | 39 | 54 |
| *Elaphe quatuorlineata* | 19 | 19 | 36 | 19 | 46 | 19 | 38 | 19 | 49 | 19 | 39 | 19 | 42 |
| *Natrix tessellata* | 187 | 92 | 110 | 46 | 67 | 43 | 70 | 36 | 41 | 36 | 58 | 33 | 39 |
| *Vipera ammodytes* | 101 | 80 | 114 | 65 | 132 | 27 | 32 | 42 | 69 | 29 | 41 | 40 | 64 |
| *Vipera berus* | 264 | 172 | 173 | 47 | 47 | 170 | 171 | 64 | 64 | 147 | 147 | 84 | 84 |
| *Vipera ursinii* | 19 | 13 | 16 | 13 | 15 | 13 | 16 | 16 | 16 | 13 | 15 | 13 | 16 |
