# Supplementary material for: Moving into Protected Areas? Setting Conservation Priorities for Romanian Reptiles and Amphibians at Risk from Climate Change
Source: PLoS One. 2013 Nov 4;8(11):e79330. doi: 10.1371/journal.pone.0079330 (PMC3855577; doi:10.1371/journal.pone.0079330)
Supplement: Table S5 — Randomisations tests of differences between mean irreplaceability scores of Natura 2000 planning units and mean irreplaceability scores of randomly selected planning units under current and future climate conditions (2020s and 2050, three emission scenarios) under limited dispersal (LimD) and no dispersal (NoD) assumptions. (DOCX) [file pone.0079330.s005.docx]

*Moving into protected areas? Setting conservation priorities for Romanian reptiles and amphibians at risk from climate change*

Viorel D. Popescu, Laurenţiu Rozylowicz, Dan Cogălniceanu, Iulian Mihăiţă Niculae, Adina Livia Cucu

**Table S5.**  Randomisations tests of differences between mean irreplaceability scores of Natura 2000 planning units and mean irreplaceability scores of randomly selected planning units under current and future climate conditions (2020s and 2050, three emission scenarios) under limited dispersal (LimD) and no dispersal (NoD) assumptions.

| **Climate scenario** | | **Natura 2000 PUs** | | **Random PUs** | | **p-value M_sim_ ≥ M_obs_** |
| --- | --- | --- | --- | --- | --- | --- |
|  |  | **mean** | **stdev** | **mean** | **stdev** |  |
| Current | | 0.319 | 0.099 | 0.238 | 0.006 | <0.001 |
| A1B2020s | NoD | 0.402 | 0.168 | 0.241 | 0.009 | <0.001 |
|  | LimD | 0.386 | 0.162 | 0.241 | 0.008 | <0.001 |
| A2A2020s | NoD | 0.380 | 0.205 | 0.238 | 0.009 | <0.001 |
|  | LimD | 0.367 | 0.196 | 0.240 | 0.008 | <0.001 |
| B2A2020s | NoD | 0.398 | 0.227 | 0.236 | 0.009 | <0.001 |
|  | LimD | 0.389 | 0.214 | 0.238 | 0.009 | <0.001 |
| A1B2050s | NoD | 0.394 | 0.247 | 0.229 | 0.009 | <0.001 |
|  | LimD | 0.351 | 0.208 | 0.237 | 0.005 | <0.001 |
| A2A2050s | NoD | 0.377 | 0.241 | 0.230 | 0.009 | <0.001 |
|  | LimD | 0.343 | 0.209 | 0.237 | 0.007 | <0.001 |
| B2A2050s | NoD | 0.372 | 0.221 | 0.232 | 0.009 | <0.001 |
|  | LimD | 0.339 | 0.177 | 0.235 | 0.007 | <0.001 |
