# Supplementary material for: Moving into Protected Areas? Setting Conservation Priorities for Romanian Reptiles and Amphibians at Risk from Climate Change
Source: PLoS One. 2013 Nov 4;8(11):e79330. doi: 10.1371/journal.pone.0079330 (PMC3855577; doi:10.1371/journal.pone.0079330)
Supplement: Figure S1 — Irreplaceability scores of planning units (10 ×10 km grid cells) for 2020s (a) and (b) and 2050s (c) and (d) under limited-dispersal (LimD) and no-dispersal (NoD) assumptions. (DOCX) [file pone.0079330.s006.docx]

*Moving into protected areas? Setting conservation priorities for Romanian reptiles and amphibians at risk from climate change*

Viorel D. Popescu, Laurenţiu Rozylowicz, Dan Cogălniceanu, Iulian Mihăiţă Niculae, Adina Livia Cucu

**Figure S1.** Irreplaceability scores of planning units (10 ×10 km grid cells) for 2020s (a) and (b) and 2050s (c) and (d) under limited-dispersal (LimD) and no-dispersal (NoD) assumptions.

**
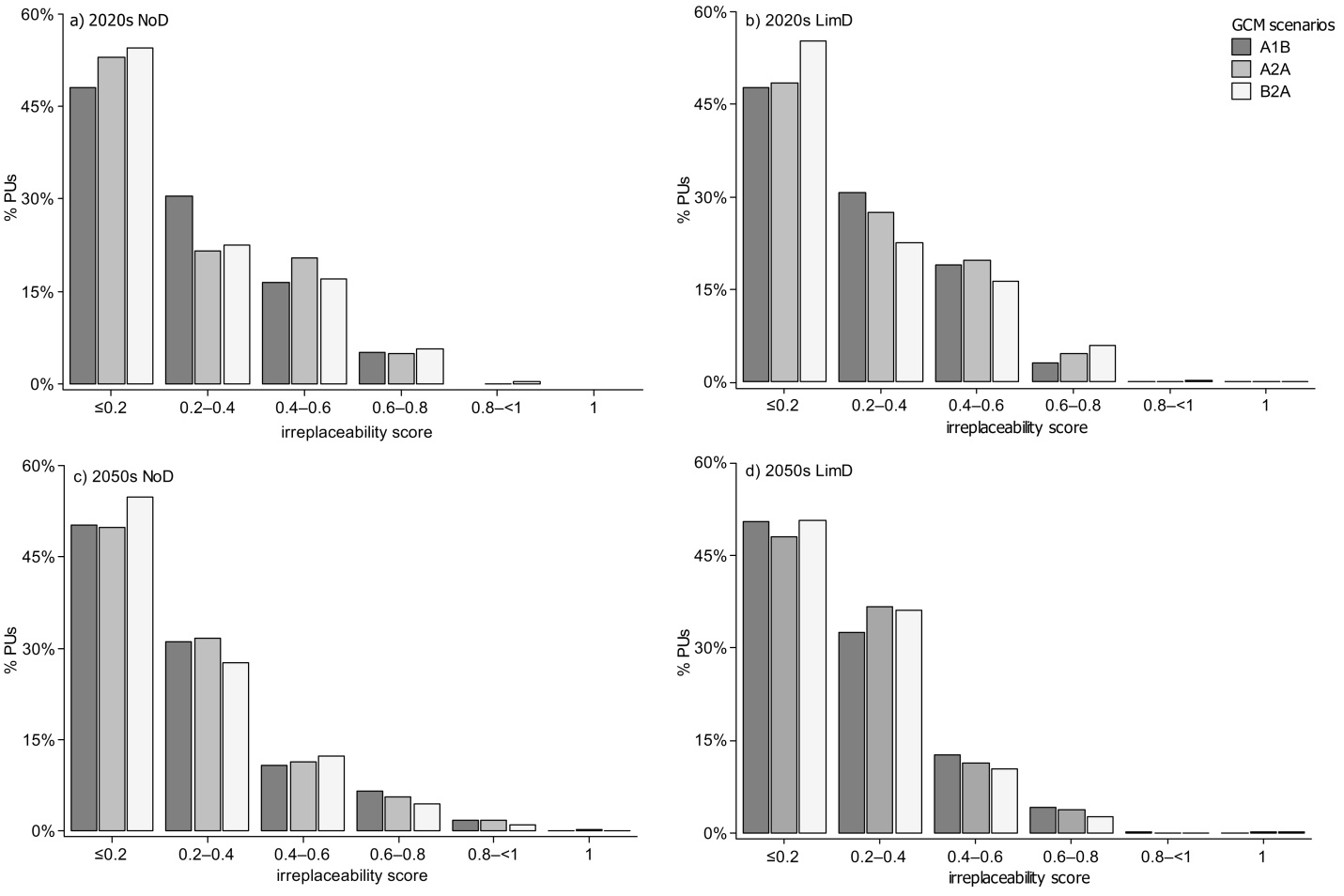
**
