# Supplementary material for: Moving into Protected Areas? Setting Conservation Priorities for Romanian Reptiles and Amphibians at Risk from Climate Change
Source: PLoS One. 2013 Nov 4;8(11):e79330. doi: 10.1371/journal.pone.0079330 (PMC3855577; doi:10.1371/journal.pone.0079330)
Supplement: Figure S2 — Future irreplaceability scores for emission scenarios A1B (a) and B2A (b) under constrained (left panels) and unconstrained (right panels) planning unit availability for the 2020s and 2050s time horizons and three emission scenarios, under limited (LimD) and no dispersal (NoD) assumptions. (DOCX) [file pone.0079330.s007.docx]

*Moving into protected areas? Setting conservation priorities for Romanian reptiles and amphibians at risk from climate change*

Viorel D. Popescu, Laurenţiu Rozylowicz, Dan Cogălniceanu, Iulian Mihăiţă Niculae, Adina Livia Cucu

**Figure S2.** Future irreplaceability scores for emission scenarios A1B (a) and B2A (b) under constrained (left panels) and unconstrained (right panels) planning unit availability for the 2020s and 2050s time horizons and three emission scenarios, under limited (LimD) and no dispersal (NoD) assumptions.

1. **
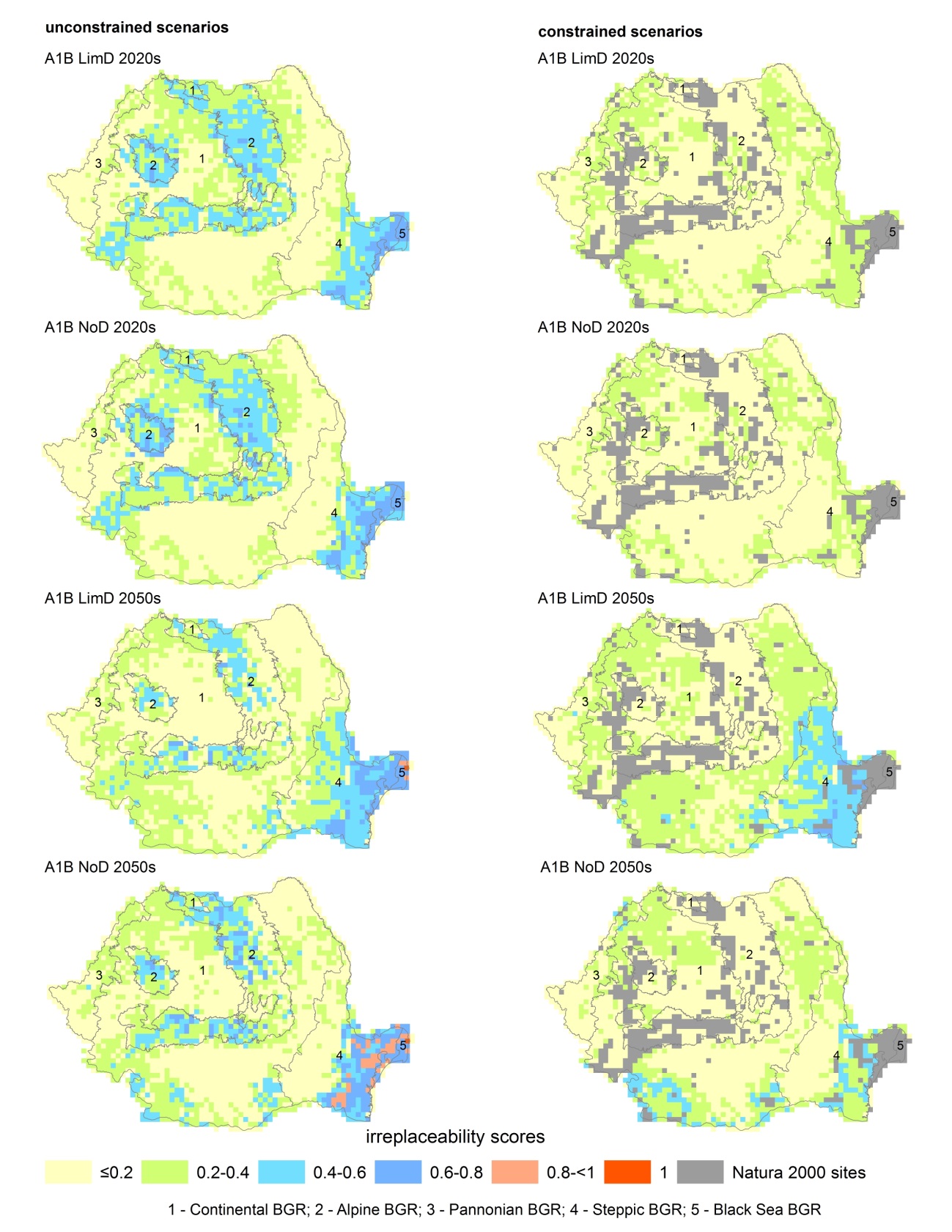
**
2. **
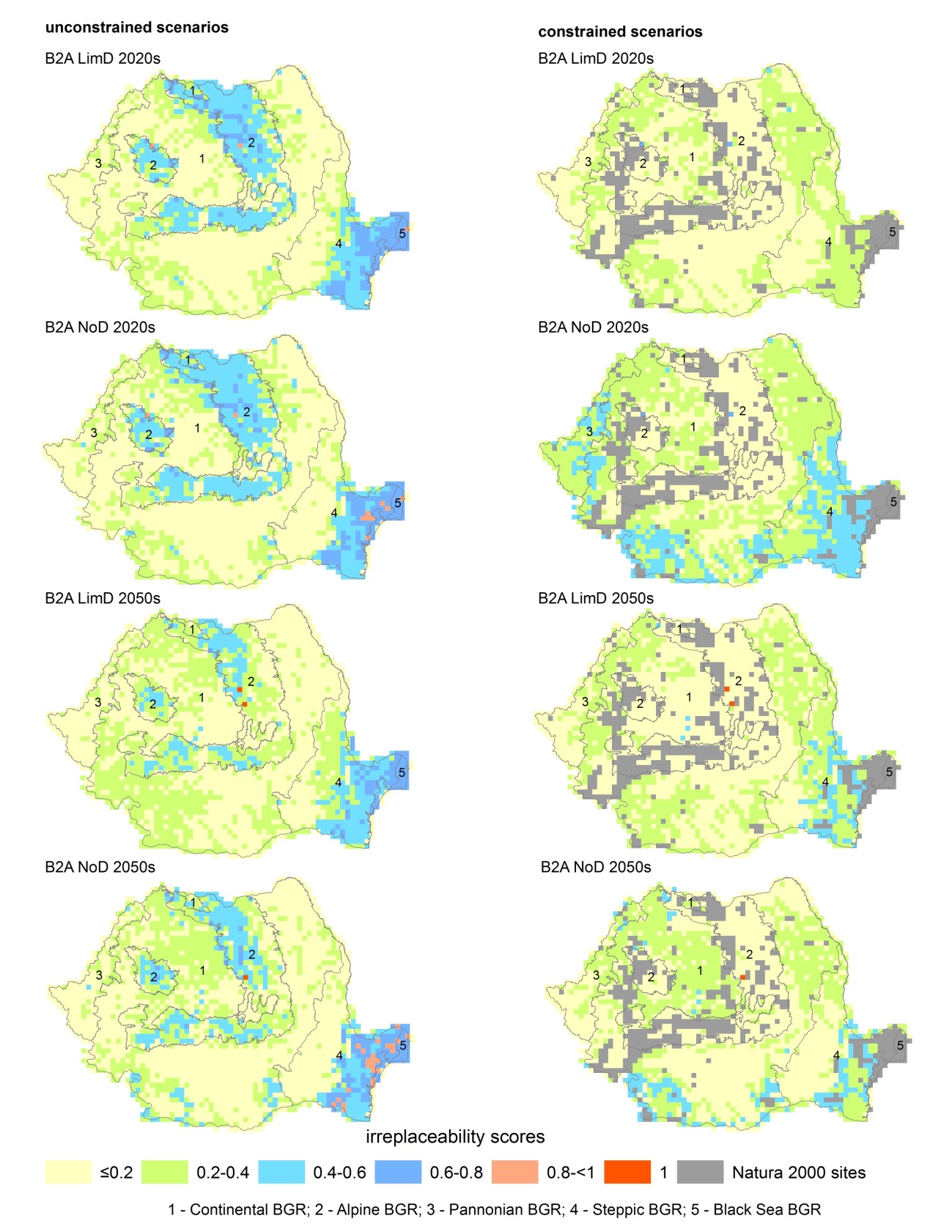
**
